# Nuclear protein FNBP4: A novel inhibitor of non-diaphanous formin FMN1-mediated actin cytoskeleton dynamics

**DOI:** 10.1016/j.jbc.2025.108550

**Published:** 2025-04-30

**Authors:** Shubham Das, Saikat Das, Amrita Maity, Sankar Maiti

**Affiliations:** Department of Biological Sciences, Indian Institute of Science Education and Research Kolkata, Mohanpur, West Bengal, India

**Keywords:** actin, FH1, FH2, FNBP4, Formin1, NLS, WW domain

## Abstract

Formin1 (FMN1), a member of the non-diaphanous formin family, is essential for development and neuronal function. Unlike diaphanous-related formins, FMN1 is not subject to canonical autoinhibition through the DID and DAD domains, nor is it activated by Rho GTPase binding. Recent studies suggest that formins also play roles in the nucleus, influencing DNA damage response and transcriptional regulation. However, the mechanisms regulating formins particularly non-diaphanous ones like FMN1 remain poorly understood. Our previous research identified the interaction between FMN1 and formin-binding protein 4 (FNBP4), prompting further investigation into its functional role in regulating actin dynamics. Results reveal that FNBP4 inhibits FMN1-mediated actin assembly *in vitro*. It is shown that FNBP4 prevents FMN1 from displacing the capping protein CapZ at the growing barbed end of actin filaments. Additionally, FNBP4 inhibits FMN1’s bundling activity in a concentration-dependent manner. Further analysis indicates that FNBP4 interacts with the FH1 domain and the interdomain connector between the FH1 and FH2 domains, creating spatial constraints on the FH2 domain. We propose that FNBP4 acts as a stationary inhibitor of FMN1. In addition, our subcellular localization studies revealed that FNBP4 is exclusively nuclear, supported by the identification of a monopartite nuclear localization signal within its sequence, suggesting a potential role in regulating nuclear actin dynamics. This study provides new insights into the regulatory role of FNBP4 in modulating FMN1-mediated actin dynamics, shedding light on regulatory mechanisms specific to non-diaphanous formins.

Actin cytoskeleton is acclaimed for its ability to shape eukaryotic cells, drive cellular movement, and facilitate numerous processes (1, 2, 3). However, its function extends beyond the cytoplasm to the nucleus, where it influences chromatin remodeling, transcription regulation, and the DNA damage repair (4, 5, 6). The nucleus contains substantial amounts of actin, both in its monomeric and filamentous forms (4). Interestingly, the actin filaments in the nucleus are generally shorter than those found in the cytoplasm (7). The nuclear cytoskeleton is orchestrated by a variety of actin-binding proteins (5, 8). Numerous actin-binding proteins and regulatory proteins, which have been extensively studied in the cytoplasm, are also present in the nucleus, including cofilin, profilin, RhoA, and some formin proteins (8, 9, 10, 11, 12). Formins, a family of actin-binding proteins, play a pivotal role in linear actin assembly through actin filament nucleation and elongation (13). In mammals, 15 formins have been identified, including mDia1, Formin 1 (FMN1), Daam1, Delphilin, and FHOD1, among others (14, 15, 16, 17, 18). Some formins have been reported to translocate to the nucleus, where they are involved in regulating actin dynamics. For instance, mDia2 continuously shuttles between the nucleus and cytoplasm using nuclear localization signal (NLS) and nuclear export signal, where it regulates transcription factor activity (19). Notably, the activity of serum response factor is regulated by the coactivator megakaryocytic acute leukemia protein, which is activated through nuclear actin assembly (20). Similarly, the FHOD1 C-terminal splice variant is localized to the nucleus, and the N-terminal FHOD1 is present in both the nucleus and cytoplasm (21). Moreover, FHOD1 interacts with Rac and facilitates actin cytoskeleton rearrangements, which are linked to the activation of serum response factor transcription (22). Previous studies indicated that under hypoxic conditions, FMN2 translocated from the cytosol to the nucleus (23). Additionally, during the DNA damage response, FMN2 accumulated in the nucleus to promote actin polymerization (24). These observations suggest that the regulation of formin activity is crucial for maintaining proper nuclear actin dynamics and ensuring nuclear homeostasis.

Mammalian formins are broadly classified into two groups as diaphanous-related formins (DRFs) and non-diaphanous formins. DRFs, such as mDia1 and Daam1, contain an N-terminal GTPase-binding domain that overlaps with the diaphanous inhibitory domain (DID), while the diaphanous autoregulatory domain (DAD) is located at the C terminal (25). DRFs remain autoinhibited through the DID–DAD interaction (26, 27). Rho GTPase binds to the GTPase-binding domain located adjacent to the DID, leading to the release of the autoinhibited state (28, 29). In contrast, non-diaphanous formins such as FMN1, FMN2, INF1, and Delphilin do not exhibit autoinhibition through the interaction of the DAD and DID domains (25). In addition to Rho GTPase, other activators, such as Rho-associated protein kinase, have been identified as key regulators of mammalian diaphanous formins like FHOD1 and mDia2 (30, 31). Moreover, Prk1 kinase has been shown to relieve the autoinhibition of the yeast formin Bni1 (32). Several formin inhibitors have also been reported, including Bud14, Smy1, Hof1, and Bil2, which inhibit the yeast formin Bnr1 (33, 34, 35, 36, 37). In mammals, inhibitors like SrGAP2 block FMNL1-driven actin filament severing, while Liprin-α3 inhibits mDia1 by competing with RhoA for binding (38, 39). Additionally, the neuronal protein Drebrin is known to inhibit mDia2 and Daam1 (40, 41). While activators and inhibitors of DRFs are well-characterized, regulators for non-diaphanous formins remain poorly understood. A recent study reported that the FMN2 N-terminal SLD domain interacts with the FH2 domain to maintain an autoinhibited conformation, which is released upon interaction with VAPA (42). Interestingly, INF2 contains DID and DAD domains, but their weak interaction is insufficient for canonical autoinhibition (43, 44). Instead, INF2 exhibits facilitated autoinhibition, wherein lysine-acetylated actin and the cyclase-associated protein complex are required for this process (44). The above-mentioned regulatory mechanisms of FMN2 and INF2 appear to be specific and do not reflect a general regulatory strategy for other non-diaphanous formins. The regulatory mechanisms of non-diaphanous formins remain largely elusive, and further research is needed to gain a more comprehensive understanding.

FMN1, a founding member of the non-diaphanous formin group. Like other formins, FMN1 contains two conserved domains: formin homology domain 1 (FH1) and formin homology domain 2 (FH2) (15, 25). FMN1 plays a crucial role in epithelial sheet formation by participating in adherence junction formation through its interaction with α-catenin, a process that does not hinder its actin nucleation function (15). Additionally, FMN1 is known to interact with microtubules *via* exon-2 (45). FMN1 is vital for various biological processes, including development, neuronal function, and cellular dynamics. For instance, FMN1 transcripts are detected in the developing kidney and the apical ectodermal ridge of the limb bud, and mutations can lead to defects such as kidney aplasia and abnormalities in limb development (46, 47, 48). In rat testes, FMN1 is involved in the formation of ectoplasmic specialization by promoting actin filament nucleation and bundling (49). Furthermore, FMN1 plays a role in dendritogenesis and synaptogenesis in mouse hippocampal neurons, as shown by the induction of these processes by Neurogenin3 (50). Deficiency of FMN1 isoform IV leads to defects in cell spreading and focal adhesion formation (51). Despite its critical functions in development and neuronal activity, the spatial regulation of FMN1 remains inadequately understood. Our previous research characterized the interaction between the WW1 domain of the formin-binding protein 4 (FNBP4) and the FH1 domain of FMN1 (52).

Here, we aim to determine the role of the FNBP4–FMN1 interaction in the regulation of actin cytoskeleton dynamics. By employing total internal reflection fluorescence (TIRF) microscopy and pyrene-actin polymerization assays, our findings demonstrate that FNBP4 inhibits FMN1-mediated actin assembly *in vitro*. Additionally, in actin filament elongation assays, we observed that FNBP4 prevents FMN1 from displacing the capping protein CapZ at the growing barbed end. Our findings also indicate that FNBP4 inhibits the bundling activity of FMN1 in a concentration-dependent manner. Moreover, our molecular docking study and surface plasmon resonance (SPR) data provide compelling evidence that FNBP4 interacts with the FH1 domain and the interdomain connector between the FH1 and FH2 domains of FMN1. In brief, this study provides novel insights into the regulatory role of FNBP4 in FMN1-mediated actin dynamics, identifying FNBP4 as a stationary inhibitor of FMN1. Subcellular localization analyses reveal that FNBP4 is exclusively nuclear, supported by the identification of a monopartite nuclear localization signal (NLS) within its sequence. These findings suggest a potential role for FNBP4 in regulating nuclear actin dynamics. Collectively, our results underscore the broader regulatory capacity of WW domain-containing proteins in actin cytoskeleton remodeling, particularly through their interactions with non-diaphanous formins.

## Results

### FNBP4 inhibits FMN1-driven actin assembly *in vitro*

**Figure 1:**
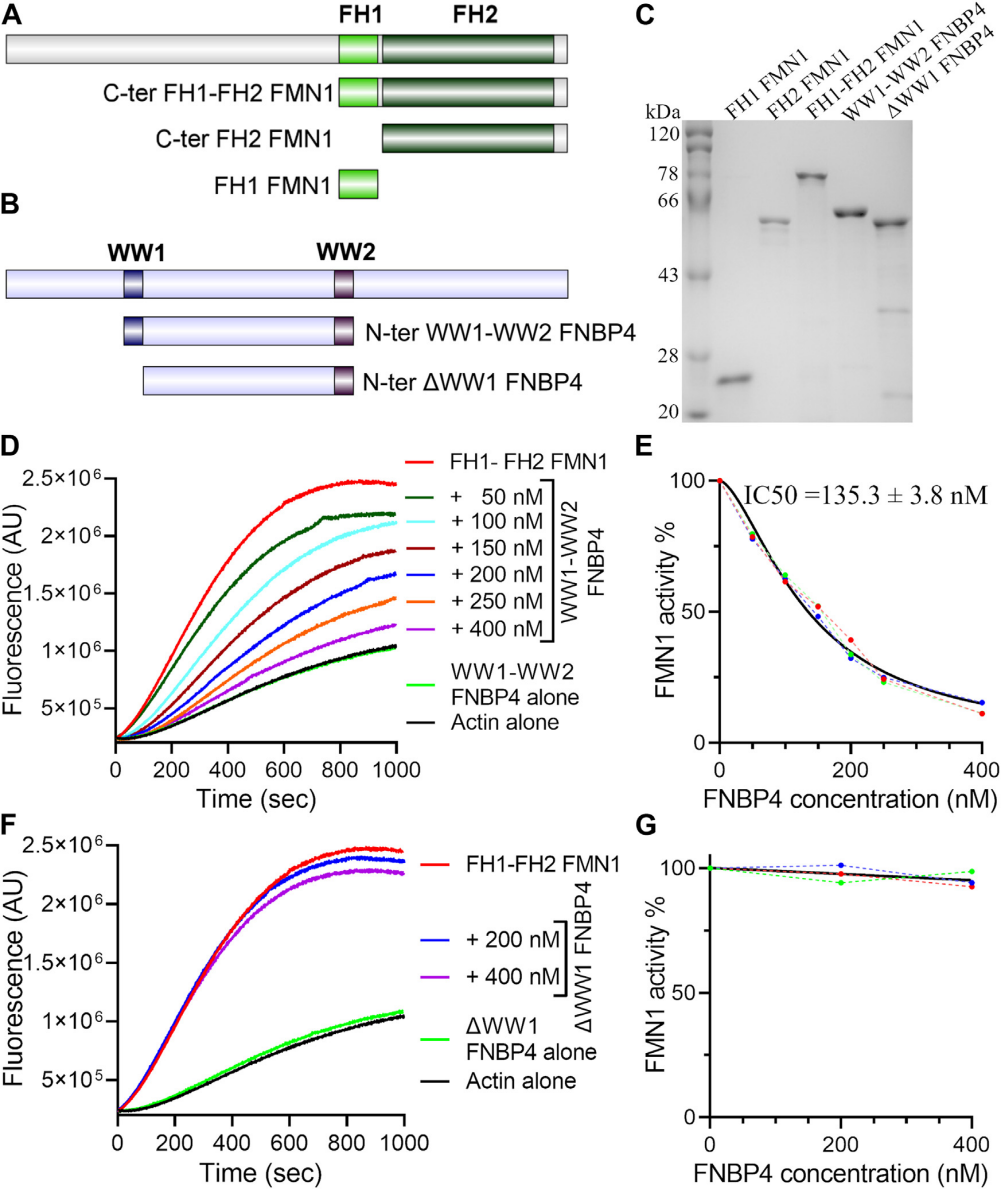
**FNBP4 is an inhibitor of FMN1.** Schematic illustrations of purified constructs of FMN1 (*A*) and FNBP4 (*B*) used for invitro experiments. Purified N-terminal WW1-WW2 FNBP4, ΔWW1 FNBP4, C-ter FH1-FH2 FMN1, C-ter FH2 FMN1, and FH1 FMN1 constructs analyzed by Coomassie-stained 10% SDS-PAGE (*C*). Pyrene-actin polymerization assay (2 μM actin monomer, 10% pyrene labeled): Actin monomers were polymerized in the presence of 50 nM FH1-FH2 FMN1 and/or increasing concentrations of N-terminal WW1-WW2 FNBP4 (*D*), and (*F*) 50 nM FH1-FH2 FMN1 and/or increasing concentrations of N-terminal ΔWW1 FNBP4. Concentration-dependent inhibitory effects of N-terminal WW1-WW2 FNBP4 (*E*) and N-terminal ΔWW1 FNBP4 (*G*) on FH1-FH2 FMN1 under the conditions outlined in (*D*) and (*F*), respectively. Percentage of FH1-FH2 FMN1 activity was quantified by dividing the slope of the actin polymerization curve in the presence of N-terminal WW1-WW2 FNBP4 or ΔWW1 FNBP4 to the slope of the curve obtained in the absence of N-terminal WW1-WW2 FNBP4 or ΔWW1 FNBP4. Actin polymerization rates were measured from the slopes of the curves between 200 and 300 s and the IC50 value was calculated as the concentration at which the polymerization rate was inhibited by 50%.

To investigate the functional consequences of the interaction between FNBP4 and FMN1 in actin cytoskeleton dynamics, we utilized various FMN1 constructs (Fig. 1*A*) and FNBP4 constructs (Fig. 1*B*). N-terminal WW1-WW2 FNBP4, N-terminal ΔWW1 FNBP4 and C-terminal FH1-FH2 FMN1 were expressed and purified from a bacterial system (Fig. 1*C*). We evaluated the effect of N-terminal WW1-WW2 FNBP4 and N-terminal ΔWW1 FNBP4 constructs on actin polymerization facilitated by FH1-FH2 FMN1 using bulk pyrene-actin assembly assays. The N-terminal WW1-WW2 FNBP4 significantly inhibited the acceleration of actin polymerization driven by FH1-FH2 FMN1 (Fig. 1*D*). N-terminal WW1-WW2 FNBP4 displayed potent, concentration-dependent inhibition of FH1-FH2 FMN1 activity, with half-maximal inhibition (of FH1-FH2 FMN1 at 50 nM) observed 135.3 ± 3.8 nM (Fig. 1*E*). In contrast, the N-terminal ΔWW1 FNBP4 construct did not influence the actin polymerization mediated by FH1-FH2 FMN1, highlighting the essential role of the WW1 domain in this regulation (Fig. 1, *F* and *G*). Notably, neither N-terminal WW1-WW2 FNBP4 nor ΔWW1 FNBP4 affects the polymerization of actin alone (Fig. 1, *D* and *F*). Furthermore, we also confirmed that WW1-WW2 FNBP4 did not bind to actin by cosedimentation assay (Fig. S1). These findings suggest that WW1-WW2 FNBP4 inhibits actin assembly by interacting with FH1-FH2 FMN1 rather than directly with actin. This aligns with our previous *in vitro* binding experiments, which demonstrated that WW1-WW2 FNBP4 exclusively interacts with FH1-FH2 FMN1, while the absence of the WW1 domain in ΔWW1 FNBP4 abolishes this interaction.

**Figure 2:**
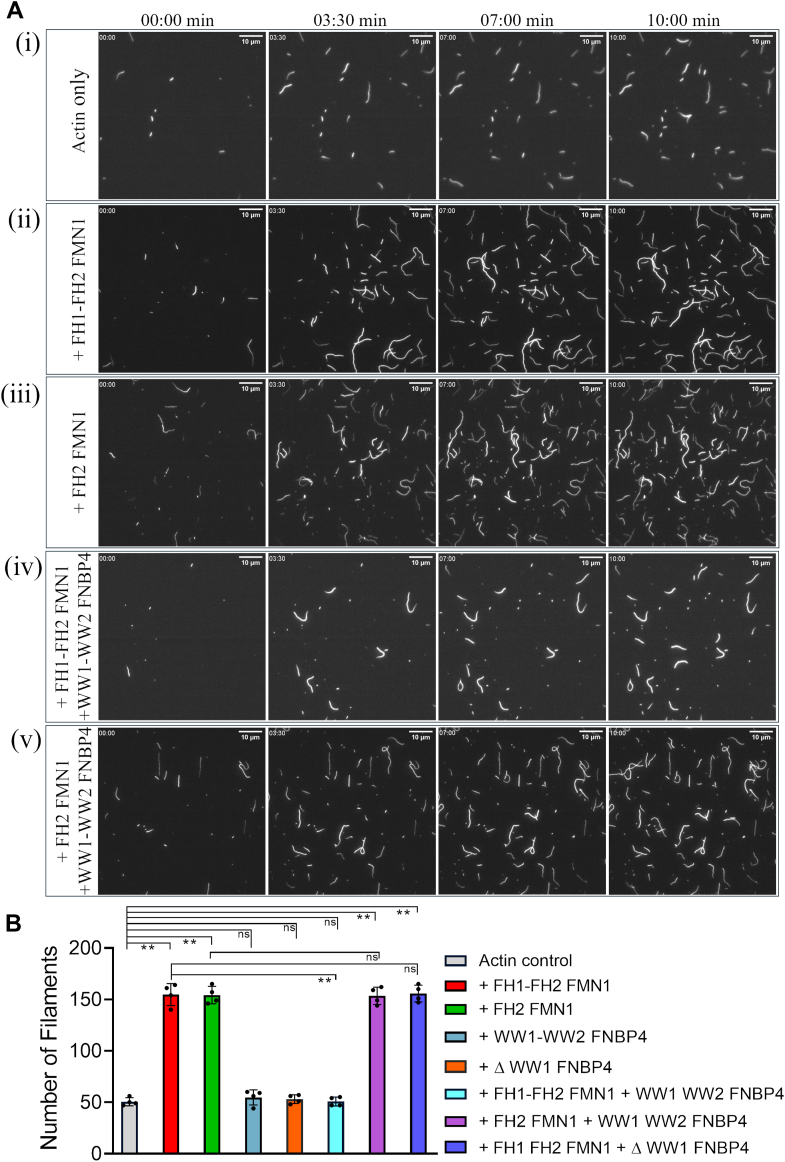
**N-terminal WW1-WW2 FNBP4 inhibits FH1-FH2 FMN1-mediated actin nucleation, observed by TIRF microscopy.***A*, time-lapse microscopy images of actin filament assembly under the conditions specified in (i) actin only, (ii) with 50 nM FH1-FH2 FMN1, (iii) 50 nM FH2 FMN1, (iv) with 50 nM FH1-FH2 FMN1 and 400 nM N-terminal WW1-WW2 FNBP4, and (v) with 50 nM FH2 FMN1 and 400 nM N-terminal WW1-WW2 FNBP4. The scale bar represents 10 μm. *B*, quantification of the number of filaments at the end point was performed in four different fields. Error bars represent the standard deviation. Statistical significance was determined using a one-way ANOVA with *post hoc* Tukey HSD test. Significance levels are denoted as ∗∗ for *p* < 0.01 and ns (not significant). HSD, honestly significant difference; TIRF, total internal reflection fluorescence.

We employed TIRF microscopy to directly observe the inhibitory effect of FNBP4 on FMN1-mediated F-actin nucleation. In this assay, monomeric G-actin was incubated with FMN1 in the presence or absence of FNBP4 (Figs. 2 and S2). When actin was incubated with either the FH1-FH2 FMN1 or FH2 FMN1, the number of actin filaments increased significantly compared to actin control (Fig. 2, *A* i, ii, iii, and *B*; Videos S1–S3). Subsequently, when actin was only incubated with either the WW1-WW2 or ΔWW1-FNBP4, the filament numbers remained comparable to the actin only control (Fig. S2 and 2*B*; Videos S4 and S7). Notably, when the FH1-FH2 FMN1 was preincubated with the WW1-WW2 FNBP4 before the addition of G-actin, there was a significant reduction in filament number compared to FH1-FH2 FMN1 control (Fig. 2, *A* ii, iv and *B*; Videos S2 and S5). No significant decrease in filament number was observed upon preincubation of the FH2 FMN1 with the WW1-WW2 FNBP4 (Fig. 2, *A* iii, v and *B*; Videos S3 and S6). Additionally, ΔWW1-FNBP4 has no effect on FH1-FH2 FMN1-mediated F-actin nucleation (Fig. S2; Video S8). These findings are consistent with our earlier results from bulk pyrene-actin assembly assays.

### FH1 domain counteracts WW1-WW2 FNBP4-mediated inhibition of FH1-FH2 FMN1 activity

**Figure 3:**
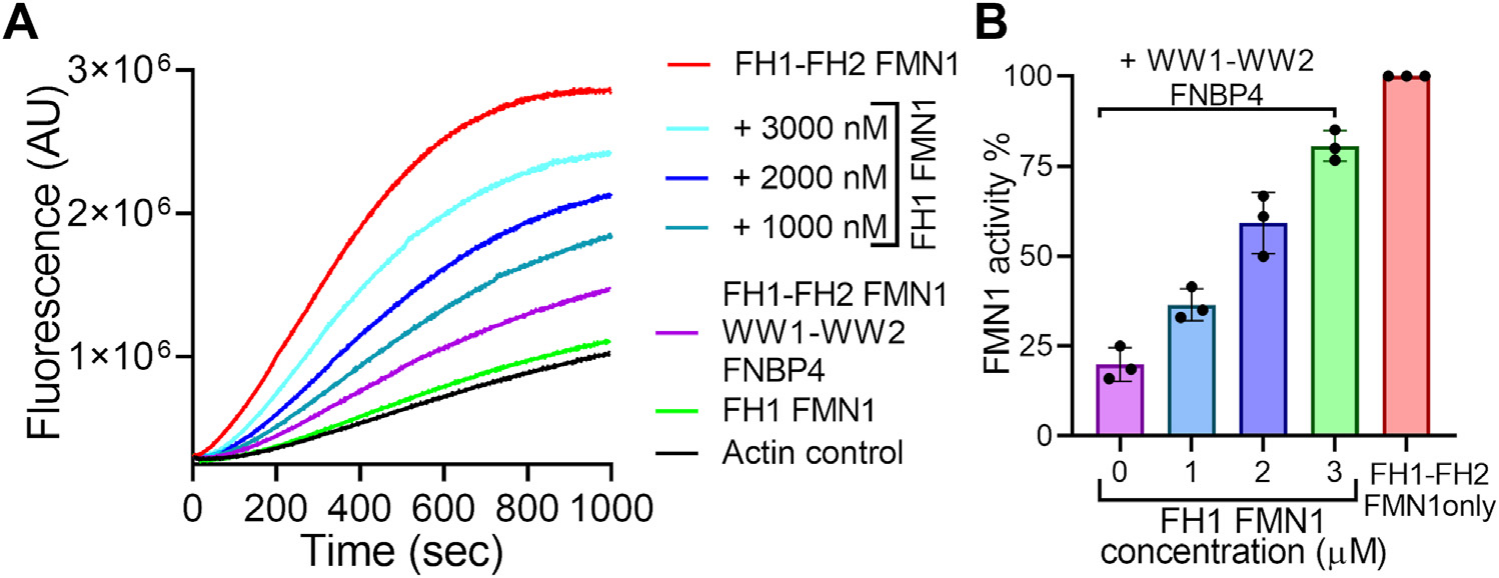
**FH1 FMN1 attenuates the inhibitory activity of WW1-WW2 FNBP4 on FH1-FH2 FMN1-mediated actin nucleation.***A*, pyrene-actin polymerization assay (2 μM actin monomer, 10% pyrene labeled) containing 50 nM FH1-FH2 FMN1 in the presence or absence of 400 nM N-terminal WW1-WW2 FNBP4, and varying concentrations of FH1-FMN1. *B*, the plot depicts the inhibition kinetics of WW1-WW2 FNBP4 on FH1-FH2 FMN1 in the presence of FH1 FMN1, where the activity of FH1-FH2 FMN1 is plotted against the concentration of FH1 FMN1. Percentage of FH1-FH2 FMN1 activity was quantified by dividing the slope (200 and 300 s) of the actin polymerization curve for N-terminal WW1-WW2 FNBP4 and FH1-FH2 FMN1, in the presence or absence of FH1 FMN1, by the slope of the curve obtained in the FH1-FH2 FMN1 control.

Then we assumed that the FH1 domain alone could counteract the inhibitory effect of the WW1-WW2 FNBP4 on FH1-FH2 FMN1-mediated actin polymerization. To investigate this, we had used a construct containing only the FH1 domain (Fig. 1, *A* and *C*) and performed an actin nucleation assay. In this assay, we incubated the FH1 domain with WW1-WW2 FNBP4, followed by the addition of FH1-FH2 FMN1 to assess its activity (Fig. 3, *A* and *B*). We observed that increasing the concentration of FH1 led to a corresponding increase in FH1-FH2 FMN1 activity. Our findings suggest that the FH1 domain alone can counter the inhibitory effect of the WW1-WW2 FNBP4 on FH1-FH2 FMN1 mediated actin polymerization. Further results suggest that the FH1 domain interacts with the ligand-binding region of the WW1-WW2 FNBP4, thereby alleviating its inhibitory effect on formin activity.

### FNBP4 modulates FH1-FH2 FMN1 activity in displacing CapZ from the barbed end of actin filaments

**Figure 4:**
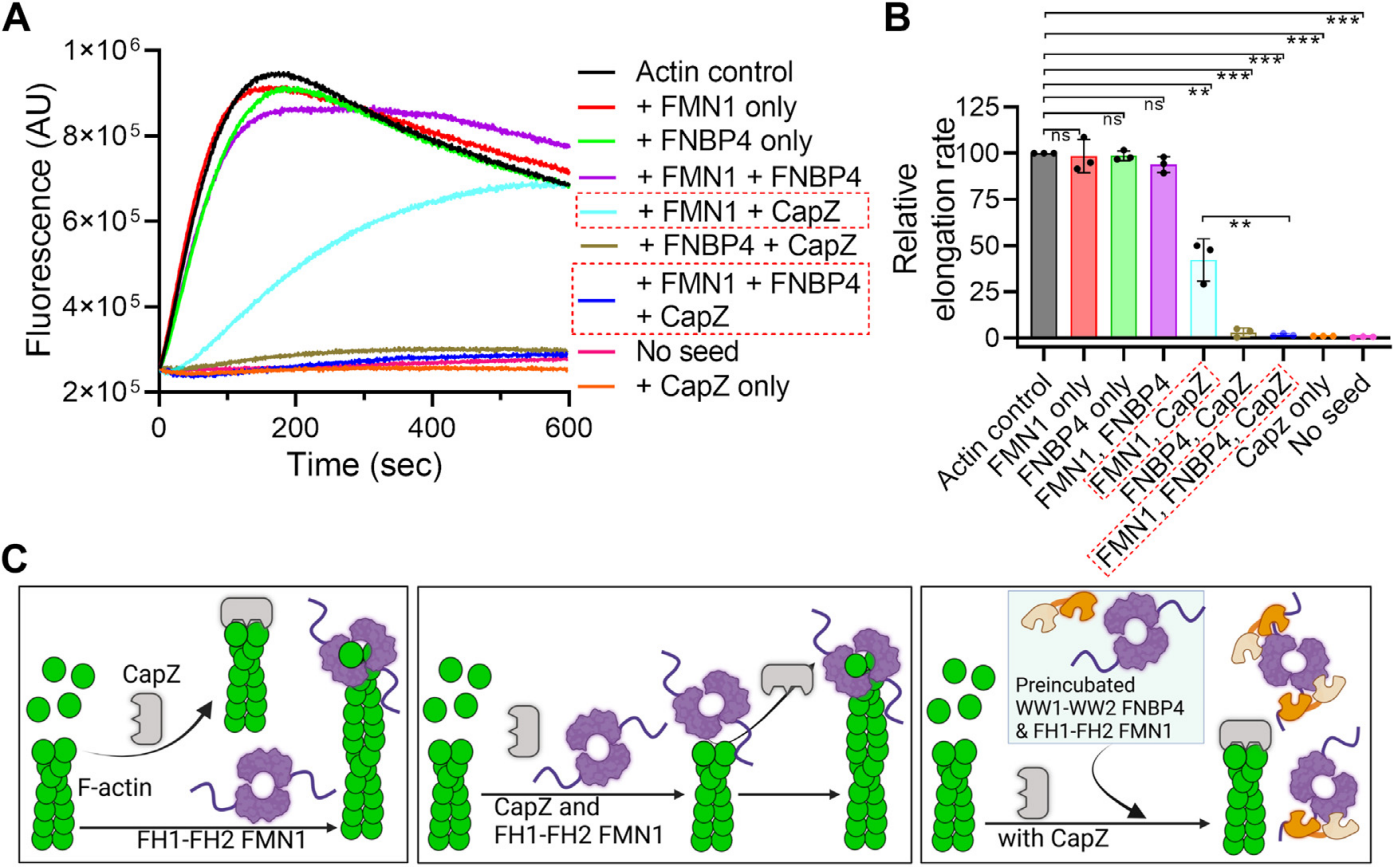
**N-ter WW1-WW2 FNBP4 inhibits FH1-FH2 FMN1 processive capping activity.***A*, effects of the N-terminal WW1-WW2 FNBP4 on FH1-FH2 FMN1 during the barbed-end growth of actin filaments, in the presence and absence of the capping protein (CapZ). An elongation assay was performed by adding actin monomers (0.5 μM, 10% pyrene-labeled) to mechanically sheared unlabeled actin seeds (333 nM) in the presence of 50 nM FH1-FH2 FMN1 and/or 400 nM WW1-WW2 FNBP4, with or without CapZ (200 nM). *B*, the plot depicts comparative analysis of the elongation rate as the slope of the fluorescence curve between 50 and 100 s of the time course, for each condition outlined in (*A*). Statistical significance was determined using an unpaired two-tailed Student's *t* test in GraphPad Prism 8. Significance levels are denoted as ∗ (*p* ≤ 0.05), ∗∗ (*p* ≤ 0.01), ∗∗∗ (*p* ≤ 0.001), ∗∗∗∗ (*p* ≤ 0.0001), and ns (not significant). *C*, a schematic representation showing the role of WW1-WW2 FNBP4 and FH1-FH2 FMN1 interaction in actin filament elongation. In the first scenario, both CapZ and FH1-FH2 FMN1 can bind to the barbed end of actin filaments. In the second scenario, FH1-FH2 FMN1 has the ability to displace CapZ from the barbed end. The third scenario illustrates that when FH1-FH2 FMN1 is preincubated with WW1-WW2 FNBP4, FH1-FH2 FMN1 cannot displace CapZ from the barbed end of the actin filament.

Biochemical studies have long established that formin and actin capping protein CapZ exert antagonistic effects on the barbed ends of actin filaments. To investigate whether FMN1 can displace CapZ from the barbed end of actin filaments, we conducted a seeded actin filament elongation assay. Expectedly, when CapZ was added to the reaction, a marked decrease in pyrene-actin incorporation at the barbed end was observed, suggesting that CapZ tightly bound to the barbed end and inhibited filament elongation (Fig. 4, *A* and *B*). In the presence of FH1-FH2 FMN1 does not significantly affect the elongation rate compared to the actin control. In contrast, the presence of FH1-FH2 FMN1 with CapZ significantly increased pyrene-actin incorporation at the barbed end compared to the CapZ control, indicating that FH1-FH2 FMN1 effectively displaced CapZ from the barbed end of actin filaments (Fig. 4, *A* and *B*). These findings indicate that FH1-FH2 FMN1 exhibits processive capping activity by displacing CapZ from the barbed end.

We then examined whether FNBP4 affects the ability of FH1-FH2 FMN1 to displace CapZ from the barbed end. To test this, we performed a similar experiment with slight modifications, FH1-FH2 FMN1 was first incubated with WW1-WW2 FNBP4, then added to the reaction, and pyrene–actin incorporation into filaments was monitored. From our observed results, the preincubated mixture of FNBP4 and FMN1 had no effect on filament elongation. In contrast, when the above incubated mixture was added to the CapZ-containing actin filament elongation mixture, the activity observed was similar to that of the CapZ only mixture (Fig. 4, *A* and *B*). These observations suggest that, in the presence of WW1-WW2 FNBP4, FH1-FH2 FMN1 is unable to displace CapZ from the barbed end (Fig. 4*C*).

### FNBP4 suppresses FH1-FH2 FMN1-driven actin bundling activity

Formins, including FMN1, are known to bundle actin filaments. Previous studies have identified FMN1 as an actin bundler, with F-actin formed by FMN1 rapidly bundling to create apical ectoplasmic specializations during spermatogenesis (49). However, earlier assessments of actin bundling were conducted using cell lysates overexpressing FMN1, other proteins present in the lysate could have influenced the observed activity. To address this, we biochemically assessed the actin bundling activity of FMN1. We further evaluated the bundling activity of the FH2 FMN1 construct *in vitro* using low-speed centrifugation assays, which revealed that FMN1 exhibits actin bundling activity in a concentration-dependent manner, confirming its previously documented bundling ability (Fig. S3).

**Figure 5:**
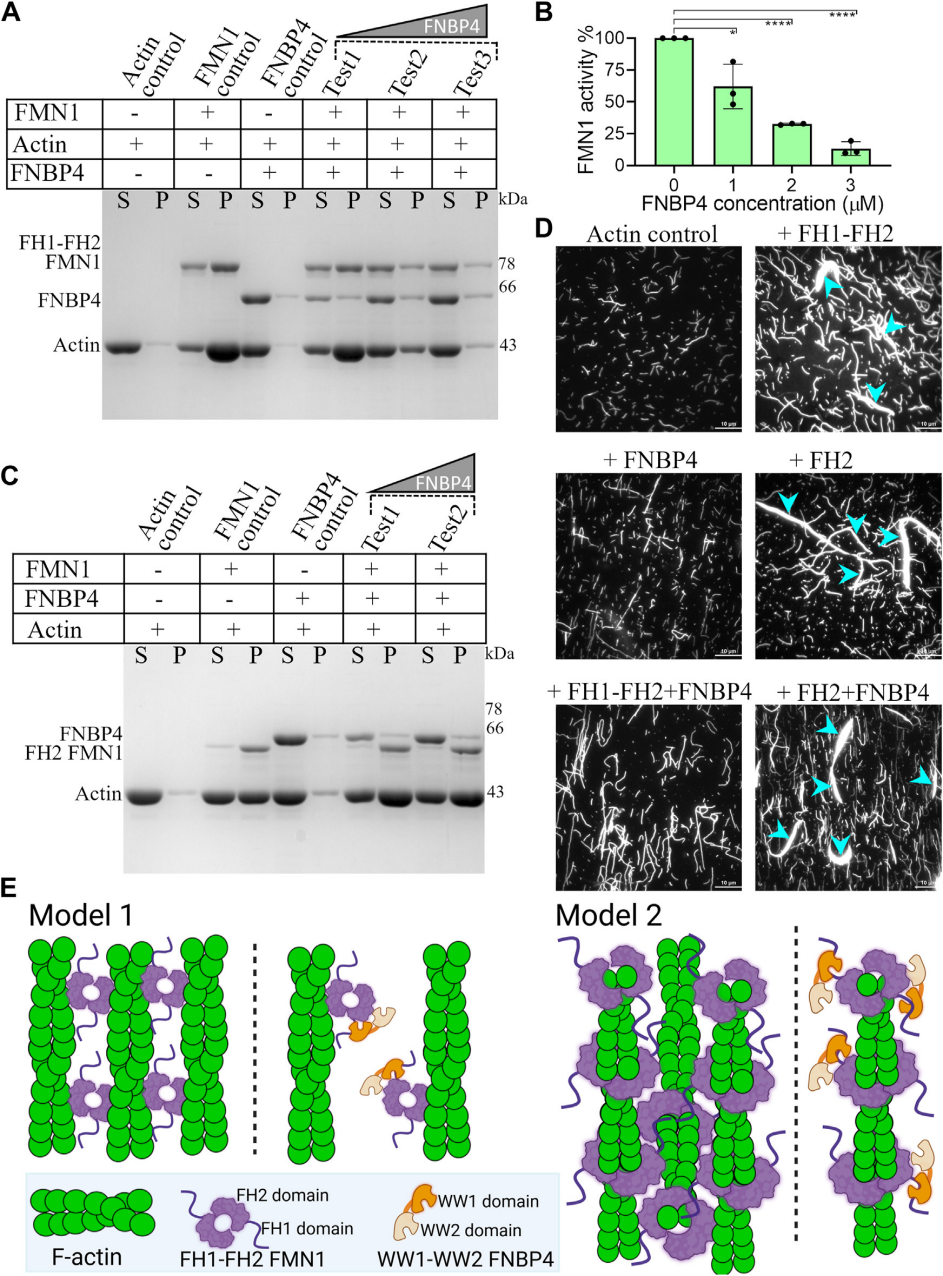
**N-terminal WW1-WW2 FNBP4 inhibits FH1-FH2 FMN1-driven actin bundling.** Low-speed centrifugation assay containing preformed 5 μM F-actin and 1 μM of FH1-FH2 FMN1 (*A*) or 1 μM of FH2 FMN1 (*C*), with increasing concentrations of WW1-WW2 FNBP4. The supernatant (S) and pelleted (P) fractions were collected and analyzed by Coomassie-stained 10% SDS-PAGE. Pellets were concentrated 5-fold for better visualization. *B*, the plot depicts the band intensity of the actin bundle pellet from FH1-FH2 FMN1 *versus* WW1-WW2 FNBP4 concentrations, as shown in panel *A*. Statistical significance was determined using an unpaired two-tailed Student's *t* test in GraphPad Prism 8. Significance levels are denoted as ∗ (*p* ≤ 0.05), ∗∗ (*p* ≤ 0.01), ∗∗∗ (*p* ≤ 0.001), ∗∗∗∗ (*p* ≤ 0.0001), and ns (not significant). *D*, TIRF microscopy images of actin bundling assay. 2 μM F-actin was incubated with FH1-FH2 FMN1 or FH2 FMN1 in the presence or absence of WW1-WW2 FNBP4 and stained with rhodamine–phalloidin. The reaction was immediately diluted 8-fold with TIRF buffer and visualized using a TIRF microscope. Scale bar is 10 μm. *E*, a schematic illustration depicting two possible mechanisms by which the bundling activity of FH1-FH2 FMN1 is inhibited *via* interaction with WW1-WW2 FNBP4. In Model 1, the first panel shows that the dimeric FH2 domain causes bundling of actin filaments by interacting with the sides of the filaments while binding to other filaments *via* its outside surface. In contrast, the first panel of Model 2 shows that the FH2 domain causes actin filament bundling by binding to filaments in a similar manner to how it binds to the barbed ends, while interacting with other filaments *via* its outside surface. The second panel of both Models 1 and 2 shows that interaction with WW1-WW2 FNBP4 inhibits the bundling activity of FH1-FH2 FMN1.

To examine whether the functional interaction between FNBP4 and FMN1 influences the actin bundling activity of FMN1, we conducted a low-speed cosedimentation assay (Fig. 5, *A* and *C*). We observed that F-actin alone did not bundle, nor did FNBP4 alone induce bundling. However, FH1-FH2 FMN1 significantly bundled actin filaments, but in the presence of WW1-WW2 FNBP4, the bundling activity was inhibited in a concentration-dependent manner (Fig. 5, *A* and *B*). This inhibitory effect was specific to FH1-FH2 FMN1 and did not affect FH2 FMN1's bundling activity (Fig. 5*C*).

Further validation was provided by TIRF microscopy. F-actin alone did not form bundles, and both FH1-FH2 FMN1 and FH2 FMN1 alone were sufficient to bundle actin filaments (Fig. 5*D*). Expectedly, when both FH1-FH2 FMN1 and WW1-WW2 FNBP4 were present, the FH1-FH2 FMN1-driven bundling activity was diminished. However, when FH2 FMN1 and WW1-WW2 FNBP4 were combined, FH2 FMN1 retained its normal bundling activity. These results suggest that WW1-WW2 FNBP4 interact with high affinity to the FH1 domain, thereby hindering the bundling activity of FH1-FH2 FMN1 (Fig. 5*E*).

### FNBP4 modulates FMN1 activity through spatial constraints on the FH2 domain

To gain dynamic insights into the interaction between FNBP4 and FMN1, we performed molecular dynamics (MD) simulation studies. The 3D structures of full length FNBP4 and FMN1 were predicted using AlphaFold Colab. The predicted structures are further validated by the predicted aligned error (PAE) heatmap, which helps assess the accuracy of interdomain predictions (53). In the PAE heatmap, blue tiles indicate regions with low error and high prediction confidence, while red tiles represent areas with higher error and lower confidence. For FMN1, the large blue region in the PAE plot corresponds to the FH2 domain, suggesting high confidence in its predicted structure (Fig. S4*A*). Similarly, for FNBP4, two prominent blue regions are observed in the PAE plot, corresponding to the WW1 and WW2 domains, indicating accurate predictions for these domains (Fig. S4*B*). With confidence in the predicted structures of FMN1 and FNBP4, we proceeded to investigate additional regulatory features of FMN1. Given that FMN1 and FMN2 belong to the same formin subtype, and that Wang *et al.* demonstrated the SLD domain of FMN2 mediates autoinhibition *via* interaction with the FH2 domain, we investigated whether a similar SLD domain is present in FMN1 (42). To address this, we performed pairwise sequence alignment between the N-terminal region of FMN1 (residues 1–869) and the SLD domain of FMN2 (residues 275–734) (Fig. S5*A*). The sequence alignment revealed only 21.77% identity between the FMN2 SLD domain and the N-terminal region of FMN1 (Fig. S5, *A* and *B*). Furthermore, structural superimposition of the FMN2 SLD domain with the FMN1 N-terminal region yielded an RMSD of 53.129 Å, thus confirming the lack of structural similarity (Fig. S5*C*). These results suggest that FMN1 does not contain an SLD domain and is unlikely to adopt an autoinhibited conformation similar to FMN2. However, further biochemical studies, including experimental validation, will be essential to fully understand the structural organization of the full-length FMN1 protein.

**Figure 6:**
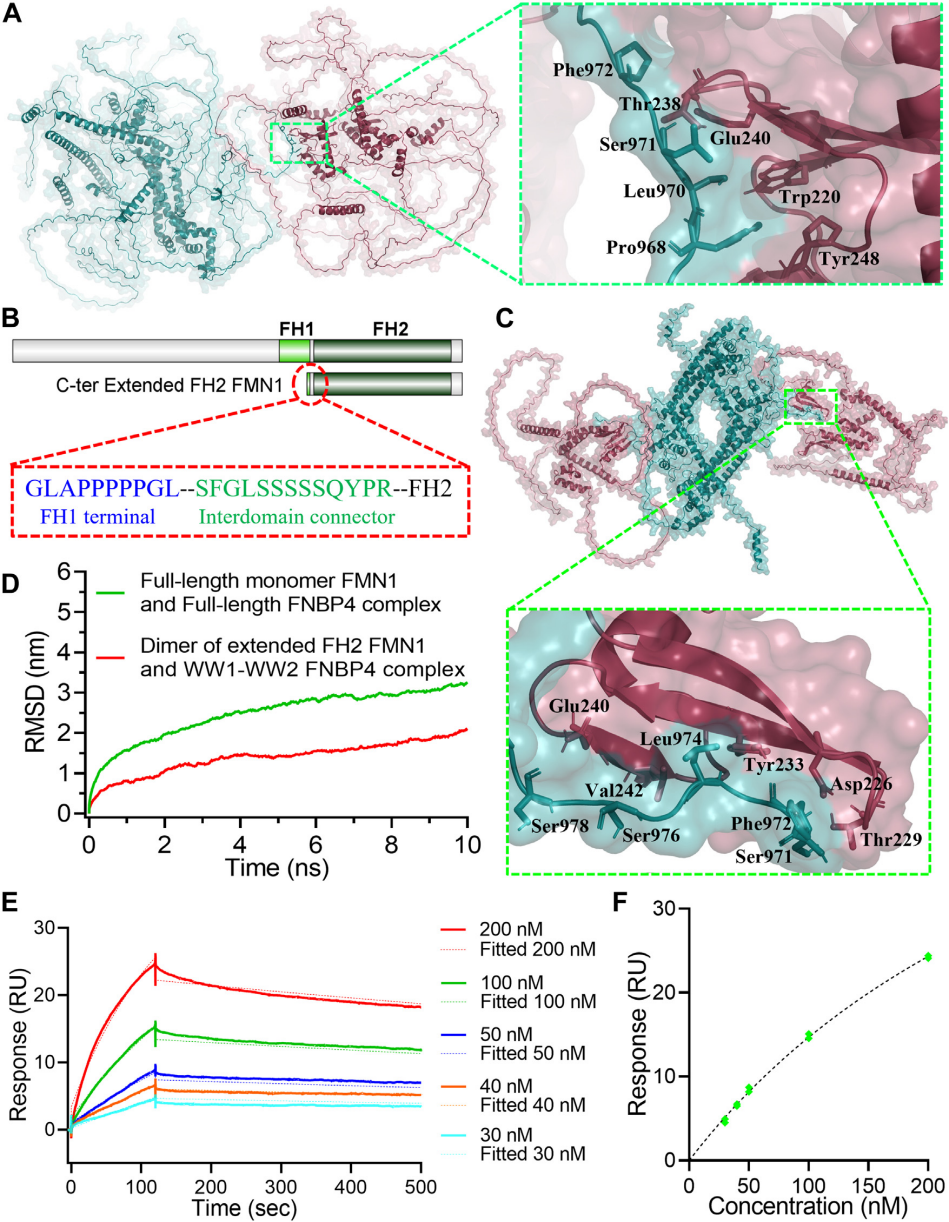
**N-terminal WW1-WW2 FNBP4 binds to FH1 domain and the interdomain connector of FMN1.***A*, the best-docked pose of the full-length monomeric FMN1 and full-length FNBP4 complex. FMN1 is shown in *teal blue*, while FNBP4 is depicted in *raspberry red*. The zoomed inset highlights the binding interface, showcasing key interacting residues critical for complex formation. *B*, a schematic representation of the domain organization of the Extended FH2 FMN1 construct, with a zoomed-in view of the terminal FH1 and interdomain connector regions, highlighting the amino acids within a *dotted red box*. *C*, a top view of the Extended FH2 FMN1 dimer in complex with two molecules of WW1-WW2 FNBP4. Extended FH2 FMN1 dimer is shown in *teal blue*, while WW1-WW2 FNBP4 is shown in *raspberry red*. The inset displays the binding interface, highlighting the key interacting amino acids involved in the interaction. *D*, a plot comparing the backbone RMSD analysis between the full-length monomeric FMN1-full-length FNBP4 complex and the Extended FH2 FMN1-WW1-WW2 FNBP4 complex, calculated over the entire 10 ns MD simulation. *E*, binding kinetics of Extended FH2 FMN1 and WW1-WW2 FNBP4 analyzed through SPR sensorgrams. Colored sensorgrams illustrate the binding of varying concentrations of Extended FH2 FMN1 to immobilized WW1-WW2 FNBP4, highlighting the concentration-dependent interaction. The association and dissociation phases were monitored for 120 and 380 s, respectively. All sensorgrams were globally fitted to a 1:1 Langmuir binding model, with the fitted curves represented by *dashed lines* in the respective colors for each dataset. *F*, the plot depicts the binding affinity of Extended FH2 FMN1 and WW1-WW2 FNBP4. The curve was plotted as response units (RU) *versus* the concentration of Extended FH2 FMN1. Each concentration was analyzed in duplicate as a positive control, and a buffer with no concentration was used as the negative control. The curve was fitted using a nonlinear regression equation (one-site specific binding model). MD, molecular dynamics; SPR, surface plasmon resonance.

An analysis of the best-scored docked complex of the full-length FMN1 monomer and full-length FNBP4 using LigPlot + revealed a predominantly hydrophobic interaction network at the interface, along with a single hydrogen bond between Glu240 of FNBP4 and Leu970 of FMN1 (Fig. S6*A*). Key interacting residues from FNBP4 include Trp220, Thr238, Tyr248, and Pro246 from the WW1 domain. The residues from FMN1 involved in the interaction are Pro968, Ser971, and Phe972 from the FH1 domain, as well as residues from the interdomain connector between FH1 and FH2 (Figs. 6*A* and S6*A*).

To further assess the stability and dynamics of the FNBP4-FMN1 complex, we conducted a 10 ns MD simulation using GROMACS. The RMSD of the complex was calculated, reaching a value of 3.25 nm by the end of the simulation (Fig. 6*D*). Additionally, the radius of gyration (Rg), which measures the compactness of the complex, yielded a total Rg value of 7.43 nm (with component values of Rgx = 3.69 nm, Rgy = 6.87 nm, and Rgz = 7.04 nm) (Fig. S6*B*). Analysis of the simulation trajectory revealed that the FNBP4-FMN1 complex underwent conformational changes over the course of the 10 ns simulation (Video S9).

To further explore the role of the interdomain connector region in its interaction with WW1-WW2 FNBP4, we performed docking experiments between the Extended FH2 FMN1 and WW1-WW2 FNBP4. For this, we constructed a 3D model of the Extended FH2 FMN1 dimer (amino acids 961–1466), which includes the terminal portion of the FH1 domain (amino acids 961–970), the interdomain connector (amino acids 970–983), and the FH2 fragment (Fig. 6*B*). The PAE plot for both the Extended FH2 FMN1 dimer and WW1-WW2 FNBP4 shows high-confidence predictions for both molecules (Fig. S7, *A* and *B*). Furthermore, we performed a detailed secondary structural analysis of the WW1-WW2 FNBP4 using circular dichroism (CD) spectroscopy to validate the disordered nature predicted by the AlphaFold model. CD spectral analysis suggested that WW1-WW2 FNBP4 remains largely disordered, although the presence of β-pleated sheets and α-helices was also evident, aligning well with the AlphaFold predictions (Fig. S7, *C* and *D*). Additionally, structural superimposition of the WW1 and WW2 domains of FNBP4 with nine experimentally resolved WW domain structures from the Protein Data Bank yielded RMSD values below 2 Å, indicating a high degree of structural conservation (Supporting Information Table S1; Fig. S7, *E* and *F*). These results confirm that the predicted structures of WW1 and WW2 accurately represent the canonical WW domain fold.

Our docking results demonstrated that the Extended FH2 FMN1 dimer could bind two molecules of WW1-WW2 FNBP4 (Fig. 6*C*). Further analysis of the binding interface in the best-scored complex between the Extended FH2 FMN1 dimer and the WW1-WW2 domains of FNBP4 revealed hydrogen bonds (Ser978, Phe972, Ser971, and Lys1007 of FMN1 with Glu240, Tyr233, Asp226, Thr229, and Glu241 of FNBP4) and hydrophobic interactions (Ser977, Ser976, Leu974, and Gly973 of FMN1 with Val242, Asn228, and Trp244 of FNBP4) (Figs. 6*C* and S8*A*). These findings highlight the role of the interdomain connector region of FMN1 and the WW1 domain of FNBP4. To further examine the stability of this interaction, a 10 ns MD simulation was conducted on the Extended FH2 FMN1 dimer and WW1-WW2 FNBP4 complex (Video S10). The RMSD of the complex stabilized at 2.06 nm by the end of the simulation, indicating a stable interaction (Fig. 6*D*). Additionally, the compactness of the complex was assessed by calculating the Rg, which yielded a total Rg value of 7.36 nm (with component values of Rgx = 3.99 nm, Rgy = 6.52 nm, and Rgz = 7.06 nm) at the end of the simulation (Fig. S8*B*).

A comparative analysis of the full-length FMN1 monomer bound to FNBP4 and the Extended FH2 FMN1 dimer in complex with WW1–WW2 FNBP4 revealed that the latter exhibited a significantly lower RMSD value (2.06 nm) compared to the former (3.25 nm) (Fig. 6*D*). The Rg value for the full-length FMN1 monomer and full-length FNBP4 complex fluctuated between 7.00 nm and 7.406 nm (Fig. S6*B*), whereas the Rg range for the Extended FH2 FMN1 dimer with the WW1-WW2 FNBP4 complex was narrower, ranging from 7.20 nm to 7.36 nm (Fig. S8*B*). The lower RMSD and narrower range of Rg fluctuation indicate that the Extended FH2 FMN1 dimer and WW1-WW2 FNBP4 complex retains better structural compactness and stability during the 10 ns simulation. These results highlight that the interaction between the dimeric Extended FH2 FMN1 and the WW1-WW2 FNBP4 is significantly stronger, contributing to the enhanced stability of the complex.

To further validate our molecular simulation study, we constructed the Extended FH2 fragment of FMN1 (amino acids 961–1466) based on the previously developed Extended FH2 FMN1 molecule (Fig. 6*B*). Furthermore, we conducted an SPR-based binding assay using purified Extended FH2 construct of FMN1 (Figs. 6*E* and S9*A*). In the SPR experiment, WW1-WW2 FNBP4 was immobilized on the sensor surface (Fig. S9, *B* and *C*), and various concentrations of the Extended FH2 were flowed over it, yielding a specific binding response (Fig. 6, *E* and *F*). Kinetic analysis revealed that a K_D_ value was calculated as 36.5 nM, k_a_ 0.225 × 10^5^ M^−1^s^−1^, and k_d_ 8.029 × 10^−4^ s^−1^. These results, combined with the molecular simulation data, provide strong evidence that the WW1 domain of FNBP4 interacts with the interdomain connector site near the FH2 domain. Additionally, our molecular docking studies identified a potential interaction between lysine 1007 of the FH2 domain, located at the beginning of the FH2 core domain structure, and glutamate 241 of the WW1 domain of FNBP4 (Fig. S8*A*). This close proximity from FH2 might create spatial constraints that impact FH2 domain function.

**Figure 7:**
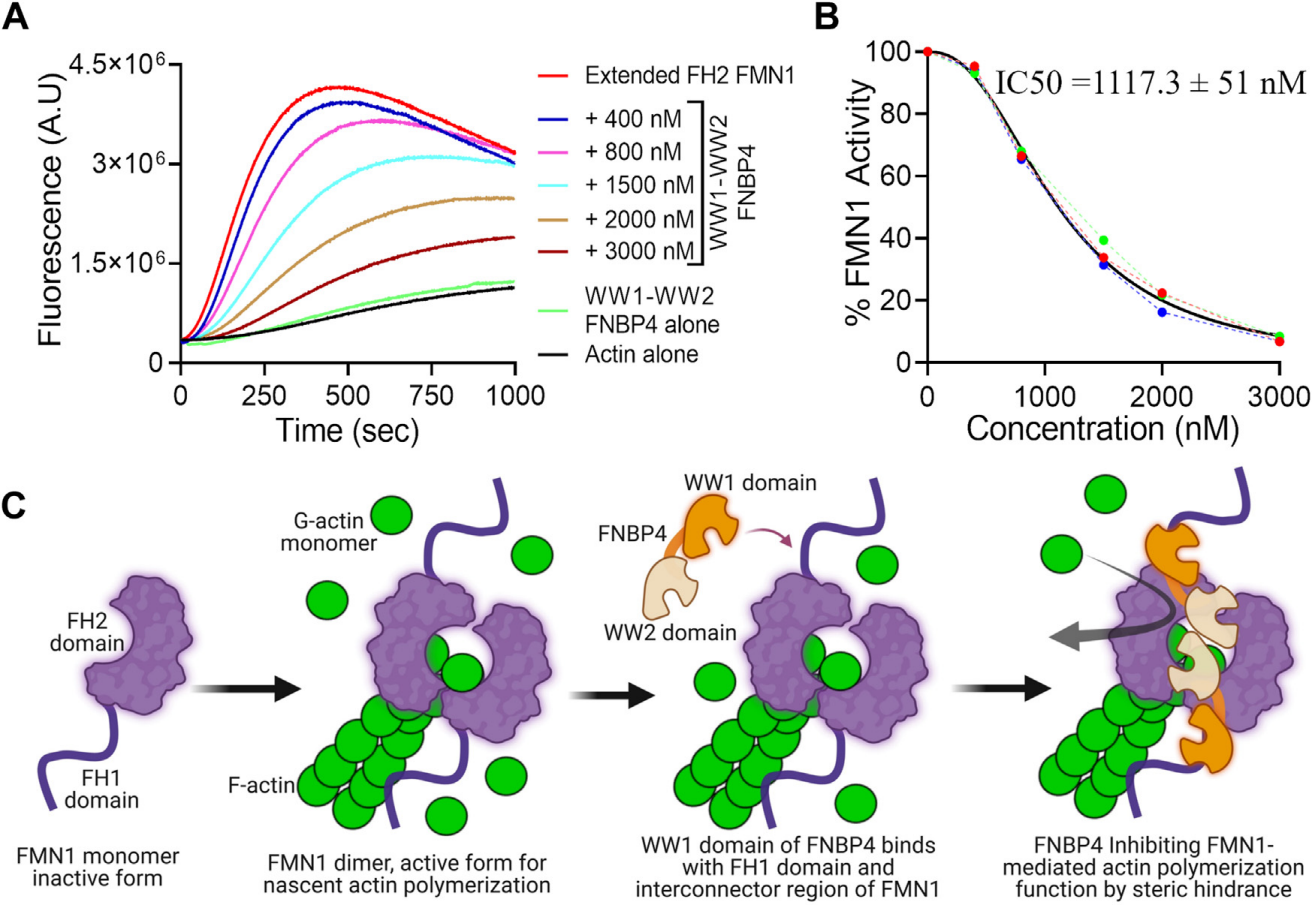
**N-terminal WW1-WW2 FNBP4 modulates FH2 domain activity through spatial constraints.** Pyrene-actin polymerization assay (2 μM actin monomer, 10% pyrene labeled): Actin polymerization was performed using 50 nM of the Extended FH2 FMN1 construct and/or increasing concentrations of the N-terminal WW1-WW2 FNBP4 (*A*). The concentration-dependent inhibitory effects of N-terminal WW1-WW2 on Extended FH2 FMN1 were observed under the same conditions outlined in (*A*). The percentage of activity of the Extended FH2 FMN1 construct was quantified by dividing the slope (200 and 300 s) of the actin polymerization curve in the presence of N-terminal WW1-WW2 FNBP4 by the slope of the curve obtained in the absence of N-terminal WW1-WW2 FNBP4 (*B*). A schematic model depicting the interaction of the WW1 domain of FNBP4 with the FH1 domain and the interdomain connector between the FH1 and FH2 domains of FH1-FH2 FMN1, which poses a steric hindrance, inhibiting its actin polymerization activity (*C*).

To test our hypothesis, we conducted a pyrene-labeled actin polymerization assay using the Extended FH2 FMN1 construct in the presence of the WW1-WW2 FNBP4. The results demonstrated a concentration-dependent inhibition of Extended FH2 FMN1, with half-maximal inhibition observed at 1117.3 ± 51 nM (Fig. 7, *A* and *B*). Additionally, we assessed the influence of the N-terminal WW1-WW2 FNBP4 on the activity of purified FH2 FMN1 (Fig. S10). Our findings suggest that WW1-WW2 FNBP4 exhibits only minimal inhibitory potency toward FH2 FMN1, in contrast to its more pronounced effect on the Extended FH2 FMN1 construct. This observation implies that WW1-WW2 FNBP4 interacts weakly and transiently with the FH2 domain of FMN1 under our experimental conditions in pyrene actin assembly assays. Consistently, our previously published SPR data did not detect a stable interaction between WW1-WW2 FNBP4 and FH2 FMN1, further supporting the notion that any binding is likely to be weak and transient. In conclusion, the inhibitory effect of WW1-WW2 FNBP4 on Extended FH2 FMN1 was weaker compared to that on FH1-FH2 FMN1. These suggest that both the FH1 domain and the interdomain connector between FH1 and FH2 play a critical role in the spatial regulation of FMN1 (Fig. 7*C*).

### Subcellular localization reveals FNBP4 as a nuclear protein

**Figure 8:**
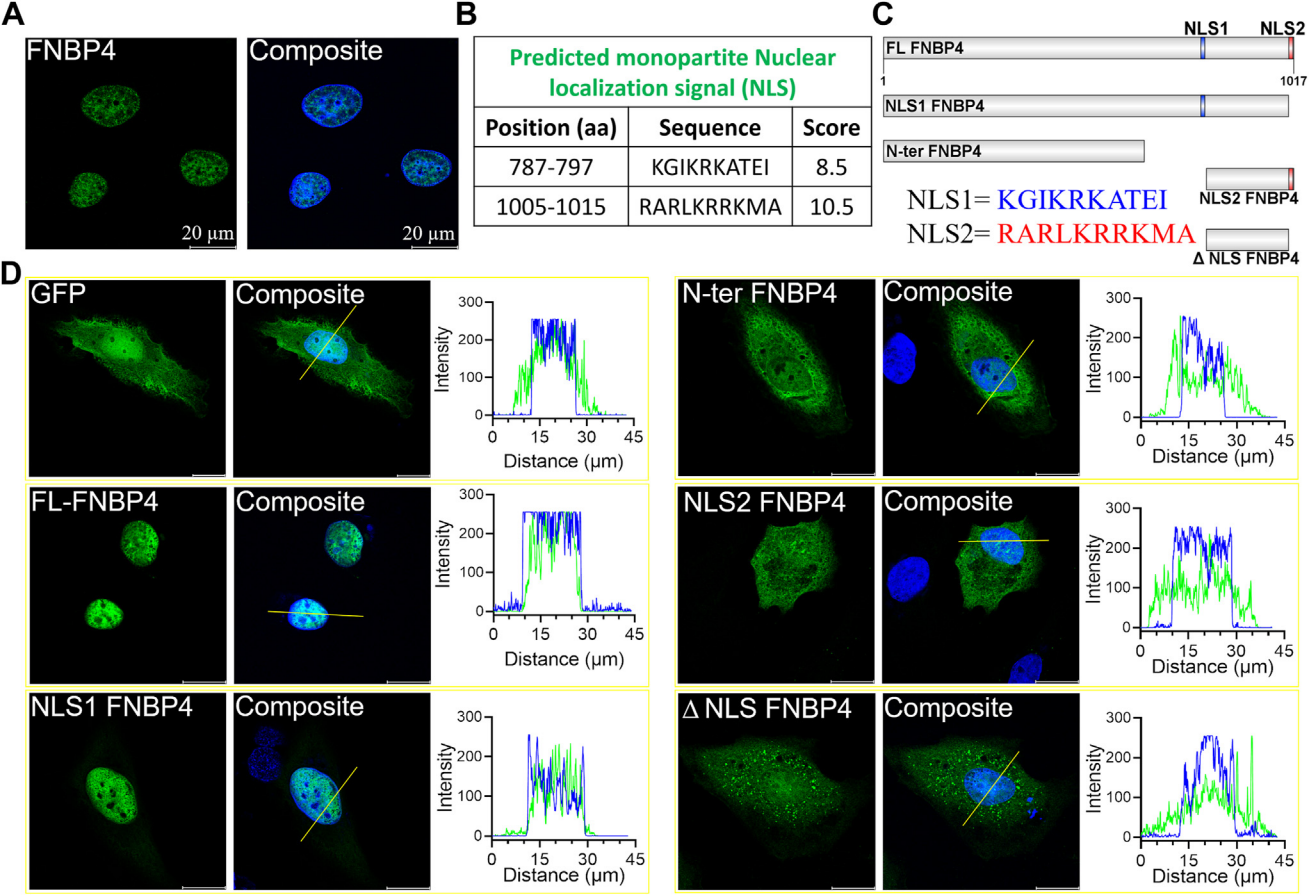
**FNBP4 contains a nuclear localization signal and localizes to the nucleus.***A*, immunofluorescence staining of FNBP4 in HeLa cells. Cells were fixed with a 1:1 mixture of ice-cold acetone and methanol. Nuclei were stained with DAPI, while FNBP4 was visualized using an anti-FNBP4 antibody. The images represent a single Z-stack section (*middle* slice). The scale bar represents 20 μm. *B*, the table show the amino acid sequences of FNBP4 with nuclear localization signal using cNLS mapper. KGIKRKATEI (787–797 aa) and RARLKRRKMA (1005–1015 aa) are predicted as monopartite nuclear localization signals. *C*, schematic representations of GFP-tagged various FNBP4 (full or truncated) constructs utilized for transient overexpression in HeLa cells. KGIKRKATEI (787–797 aa) and RARLKRRKMA (1005–1015 aa) represented as NLS1 and NLS2, respectively. *D*, representative images show subcellular distributions of GFP-tagged different truncated FNBP4 constructs in HeLa cells. HeLa cells were transiently transfected with either the vector alone or GFP-tagged FNBP4 (full or truncated) constructs, followed by fixation with PFA and DAPI staining of the nuclei. The *right*-side plot shows the fluorescence intensity profiles of GFP or GFP-tagged FNBP4 constructs and DAPI along the *white* line marked across the cytoplasm and nucleus of the respective merged image. The scale bar represents 20 μm. DAPI, 4′,6-diamidino-2-phenylindole; PFA, paraformaldehyde.

To investigate the subcellular localization of endogenous FNBP4 expression, we performed immunofluorescence staining in HeLa cells and visualized the results using confocal microscopy (Fig. 8*A*). The confocal images revealed that FNBP4 was exclusively localized in the nucleus. To further investigate the regions of FNBP4 responsible for its nuclear localization, we predicted its NLSs using the NLS predictor software. Two monopartite NLS sequences were identified, KGIKRKATEI (787–797 aa) and RARLKRRKMA (1005–1015 aa) (Fig. 8*B*). Based on these predictions, we constructed GFP-tagged full-length and truncated mutants of FNBP4 and expressed them as GFP-fusion proteins in HeLa cells to examine their localization (Fig. 8, *C* and *D*). The full-length FNBP4 (1–1017 aa) localized exclusively to the nucleus, similar to the endogenous protein (Fig. 8*D*). The C-terminal NLS2 deleted construct, NLS1 FNBP4 (1–1004 aa), also predominantly localized to the nucleus, suggesting that the NLS1 sequence (KGIKRKATEI, 787–797 aa) is sufficient to target the protein to the nucleus, even though the molecular weight exceeds 100 kDa, ruling out passive diffusion.

In contrast, the C-terminal deleted construct, N-ter FNBP4 (1–629 aa), localized to the cytoplasm. Interestingly, the NLS2 only fragment, NLS2 FNBP4 (801–1017 aa), localized to the cytosol despite having a molecular weight below 50 kDa, which is typically associated with nuclear import. A construct lacking both NLS1 and NLS2 (ΔNLS FNBP4, 801–1004 aa) also localized to the cytosol (Fig. 8*D*). These findings indicate that NLS1 is both critical and sufficient for nuclear localization, whereas NLS2 does not appear to be involved in the nuclear targeting of FNBP4.

## Discussion

We demonstrated that the WW1-WW2 FNBP4 construct inhibits FMN1-mediated actin nucleation *in vitro* (Figs. 1*D* and 2*A* iv), while the truncated ΔWW1-FNBP4 mutant does not exhibit this inhibitory activity (Figs. 1*F* and S2). Further analysis revealed that the WW1-WW2 FNBP4 construct exerts strong inhibitory effects on the FH1-FH2 FMN1 construct (Figs. 1*D* and 2*A* iv), while it has minimal effect on FH2 FMN1 (Figs. 2*A* v and S10), suggesting a functional disparity in its regulatory influence. Our previously published data also demonstrate that the WW1-WW2 domains of FNBP4 exhibit a strong affinity for the FH1-FH2 FMN1, with a dissociation constant (K_D_) of ∼2 nM, while showing no interaction with the FH2 domain alone (52). We speculate that the binding interface between FMN1 and FNBP4 may provide valuable insights into the precise mechanism underlying the inhibition of FMN1. Our molecular docking and simulation studies identified Valine 923, Proline 968, and Leucine 970 from the FH1 domain, as well as Serine 971, Serine 978, and Phenylalanine 972 from the interdomain connector between the FH1 and FH2 domains (Fig. 6*A* and S6*A*), as key residues involved in the interaction with FMN1. Additionally, MD simulation data of the dimeric Extended FH2 FMN1 and the WW1-WW2 FNBP4 complex further highlighted the importance of the interdomain connector region of FMN1, as residues Ser978, Phe972, Ser971, Ser977, Ser976, Leu974, Gly973, and Lys1007 were involved (Figs. 6*C* and S8*A*). Furthermore, our SPR data demonstrate that the interaction between the Extended FH2 FMN1 construct (comprising amino acids 961–970 of FH1, the 970–983 aa of interdomain connector, and the 983–1466 aa of FH2 domain) and WW1-WW2 FNBP4 (Fig. 6, *E* and *F*), further reinforce the involvement of these amino acids in this interaction. The affinity of the Extended FH2 FMN1 with the WW1-WW2 FNBP4 is ∼ 36.5 nM which is comparatively less than that of the FH1-FH2 FMN1 interaction. Given that the FH1 domain of FMN1 contains six polyproline tracts, it is likely that the WW1 domain binds multiple tracts, contributing to the observed high-affinity interaction (52). However, when we used the Extended FH2-FMN1 construct, which retains only a single polyproline tract thus substantial reduction in binding affinity suggests that additional polyproline stretches play a crucial role in stabilizing the interaction. Together, these findings indicate that the full-length FH1 domain enhances binding affinity, while the 961 to 983 amino acid region plays a crucial role for the interaction and contributing to the inhibitory mechanism (Figs. 6 and 7). Previous studies have identified similar inhibitory mechanisms, where inhibitors target multiple regions of formins. For instance, DrebrinA inhibits mDia2 by interacting with both the FH2 domain and the FH2 tails (40, 41). Similarly, the F-BAR and SH3 domains of Hof1 bind to the FH2 and FH1 domains of Bnr1, respectively, resulting in the inhibition of Bnr1 (35, 36). Further structural studies are needed to gain a deeper understanding of the FNBP4-mediated inhibition of FMN1.

To our knowledge, this is the first report demonstrating the regulation of a formin by a WW domain-containing protein. Interestingly, the WW1 and WW2 domains of FNBP4 appear to function differently, suggesting that WW2 may play an important role in other functions. Previous studies have shown that the polyproline-rich FH1 domain can bind to SH3 domains, WW domains, and profilin (52, 54). It is well established that the FH1 domain of formins plays a critical role in elongation by recruiting the profilin–actin complex (55, 56). Our findings indicate that formins are finely tuned for spatial regulation of cytoskeleton dynamics *via* the FH1 domain, which can act as both an accelerator and an inhibitor depending on its interaction with formin-binding proteins. Actin elongation is promoted through the recruitment of the profilin–actin complex by the FH1 domain, whereas polymerization inhibition occurs through the interaction with FNBP4 (Figs. 1 and 2). These results highlight the intriguing regulatory role of WW domain-containing proteins in actin cytoskeleton dynamics, particularly through their interactions with formins.

Several mechanisms of formin regulation have been proposed, many of which focus on the interaction of specific domains in formin-binding proteins with the formin protein itself. A notable example is the dimeric GST-Hof1-CT, which has been shown to inhibit the actin nucleation and elongation activities of Bnr1 through a proposed “restraint model” (35). In this model, SH3 domain interferes with the FH1 domain’s ability to transfer the profilin–actin complex to the FH2 domain, thus hindering actin polymerization. However, the situation is quite different for FNBP4, where no such dimerization between the WW1 and WW2 domains occurs, suggesting that this restraint model is not applicable to FNBP4-mediated inhibition of FMN1. Alternatively, the model of Bud14 displacing Bnr1 from the barbed end offers a more realistic approach for formin inhibition, but it requires a strong interaction with the FH2 domain (33). This model is not applicable to our study as the WW domains of FNBP4 did not interact strongly with the FH2 domain of FMN1.

In contrast, we hypothesize that FNBP4 acts as a stationary inhibitor of FMN1 (Fig. 7*C*). We propose that FNBP4 interacts with the FH1 domain and the interdomain connector of the FH1 and FH2. This suggests that FNBP4 is positioned very close to the FH2 domain and imposes spatial constraints that restrict FH2’s access to actin monomers or reduce its flexibility, thus inhibiting actin filament nucleation. As it is well-known, FH2 forms a ring-shaped antiparallel head-to-tail dimer *via* interactions between the lasso and post regions and remains in a closed state (57). For actin polymerization to proceed, the FH2 domain must open up to accommodate actin monomers at its binding surface. This requires the expansion of the FH2 ring by approximately 25 Å, a process facilitated by the flexible linker region, which allows unrestricted movement of the lasso (58). Studies have shown that reducing the flexibility of the linker (*e.g.*, by shortening its length) impairs FH2-mediated actin polymerization, highlighting the critical role of lasso-linker flexibility (58, 59). We speculate that the involvement of the interdomain connector and FH1 domain of FMN1 with the WW1-WW2 FNBP4 may impose steric hindrance on the lasso, limiting its flexibility (Fig. 7*C* and Video S10). This restriction could reduce the expansion of the FH2 ring, hindering its ability to accommodate sufficient actin monomers. This model aligns with observations from other formin-binding proteins, such as srGAP2, which modulates actin filament severing activity by interacting with the FH1 domain of FMNL1 (38). The SH3 domain of srGAP2 is thought to sterically hinder the FH2 domain, preventing its full activation and inhibiting filament severing (38). Similarly, we suggest that FNBP4’s interaction with FMN1 may sterically impede FH2 activity, contributing to the observed regulation of actin dynamics.

We have also deciphered that the WW1-WW2 FNBP4 inhibits processive capping activity of FH1-FH2 FMN1 (Fig. 4). Our elongation data suggested that the FNBP4-FMN1 complex could not displace CapZ from the barbed end (Fig. 4, *A* and *B*). Interestingly, the FNBP4-FMN1 complex on actin elongation has no effect compared to the elongation rate of the actin alone. These observations lead us to speculate that the FNBP4-FMN1 complex cannot bind to the barbed end (Fig. 4*C*); if binding occurred, the elongation rate would be expected to be lower compared to the actin control. Our findings contrast with the Smy1-Bnr1 complex in yeast, where Smy1 slows the elongation rate of Bnr1 without affecting its processive capping activity (34). The Smy1-Bnr1 complex binds to the barbed end, acting as a damper to reduce both nucleation and elongation rates (34). In contrast, the FNBP4-FMN1 complex does not appear to bind to the barbed end, aligning with our stationary inhibitor model and highlighting a mechanistic divergence between the two complexes (Fig. 4*C*).

Furthermore, we demonstrated that the WW1-WW2 FNBP4 inhibits the FH1-FH2 FMN1 driven F-actin bundling activity (Fig. 5, *A* and *B*). To our knowledge, this is the first report showing the inhibition of formin-driven actin bundling in presence of WW1-WW2 FNBP4 which does not directly interact with the FH2 domain (Fig. 5, *C* and *D*). This contrasts with the previously described mechanism by which DrebrinA inhibits mDia2 F-actin bundling by binding to the FH2 domain (40, 41). Therefore, our findings provide a novel mechanistic insight into the inhibition of formin-driven actin bundling through the FNBP4-FMN1 complex (Fig. 5*E*). The role of FMN1 as an actin bundler, contributing to the formation of apical ectoplasmic specializations during spermatogenesis, has been established in previous studies (49). However, the physiological significance of FNBP4's inhibition of FMN1-mediated actin bundle formation remains to be further explored.

Our bioinformatics analysis identified two putative monopartite NLS sequences in FNBP4 (Fig. 8*B*). However, GFP-tagged different FNBP4 construct overexpression data revealed that only the NLS1 sequence is sufficient to direct the protein to the nucleus, while NLS2 alone does not exhibit this capability (Fig. 8*D*). Interestingly, the functional disparity between NLS1 and NLS2 suggests that NLS2 might serve a role distinct from nuclear localization. Previous studies have reported alternative functions for NLS sequences beyond their canonical role in nuclear import, warranting further investigation into the potential noncanonical roles of NLS2 in FNBP4. While major regulators of actin cytoskeleton dynamics, such as profilin, cofilin, and Rho GTPases, are known to shuttle between the cytoplasm and nucleus (8, 10, 11). This process is facilitated by their relatively small molecular weights (below 50 kDa), allowing diffusion through the nuclear pore complex. In contrast, the theoretical molecular weight of FNBP4 is approximately 110 kDa, indicating that FNBP4 may have evolved specialized mechanisms to mediate nuclear actin dynamics, potentially through its regulation of formin. Furthermore, Chan *et al.* previously demonstrated that FMN1 localizes to the nucleus and identified it as the primary site of its action (60). *In vitro*, formins can elongate actin filaments up to 50 μm (61). However, given the nuclear diameter of approximately 10 μm and previous reports indicating the presence of much shorter filaments within the nucleus, the physiological need for formin inhibition becomes evident. This suggests that mechanisms inhibiting the active form of formins, such as FNBP4-mediated inhibition, might be essential. Notably, FNBP4 has been identified as a prognostic marker for cancer (62). Interestingly, it has been observed that enhanced nuclear F-actin accumulation and bundling can reduce cancer cell invasion while promoting apoptosis (63). In line with this, FMN1 has been reported to interact with both FBOX3 and p53, and to cooperate with mDia2 in the nucleus (64). Additionally, FMN1 has been shown to regulate the transcriptional activity of p53, suggesting its potential role in cancer and other diseases involving p53-mediated cellular processes. This implies that FNBP4 might inhibit FMN1-driven nuclear actin accumulation and bundle formation. Further studies are necessary to establish whether FNBP4 physiologically regulates FMN1 in the nucleus and promotes cancer progression by modulating nuclear actin dynamics. However, there are other questions like how FMN1 is activated or FNBP4 is released from FMN1, which are yet to be elucidated.

Notably, this is the first report of non-diaphanous formin inhibition, which is supported by the steric hindrance model. Furthermore, it gains more significance, because this regulation is speculated to occur in the nucleus, as the FMN1 inhibitor FNBP4 is solely localized in the nucleus.

## Experimental procedures

### Plasmid construct and cloning

The mouse FMN1 gene was obtained as an FMN1 plasmid (pEGFPC2-FmnIso1a) from Addgene (plasmid #19320). Additionally, human FNBP4 complementary DNA was purchased from Dharmacon (MHS6278–202758566). Subsequently, we prepared FMN1 and FNBP4 constructs as previously described in (52). Briefly, the constructs included the C-terminal FH1-FH2 (amino acids 870–1466), C-terminal FH2 (amino acids 983–1466), FH1 (amino acids 870–970), and Extended FH2 (amino acids 961–1466) of FMN1, all cloned into the pET28a(+) vector (Novagen). The N-terminal WW1-WW2 FNBP4 (amino acids 214–629) and ΔWW1 FNBP4 (amino acids 249–629) constructs were also inserted into the pET28a(+) vector. Based on two predicted NLS sequences of FNBP4, KGIKRKATEI (amino acids 787–797) and RARLKRRKMA (amino acids 1005–1015), we cloned GFP-tagged constructs of FNBP4, including full-length FNBP4 (amino acids 1–1017), NLS1 FNBP4 (amino acids 1–1004), N-ter FNBP4 (amino acids 1–629), NLS2 FNBP4 (amino acids 801–1017), and ΔNLS FNBP4 (amino acids 801–1004) into the pEGFP-C1 vector. The clone constructs of the capping protein mouse CapZ α1 and β2 subunits in the pET-28a(+) vector were previously described (65).

### Protein expression and purification

FMN1 and FNBP4 clone constructs were expressed in BL21 (DE3) *Escherichia coli* cells. The cultures were cultivated to the log phase (*A*_600_ = 0.5) at 37 °C and then induced with 0.5 mM IPTG. FMN1 expression was induced for 12 h at 18 °C, while FNBP4 expression was induced for 8 h at 25 °C. The bacterial cells were harvested by centrifugation and subsequently lysed by sonication in lysis buffer containing 0.2% IGEPAL, 150 mM NaCl, 30 mM imidazole (pH 8), 0.5 mM DTT, and 50 mM Tris (pH 8), along with a protease inhibitor cocktail (Aprotinin, Pepstatin A, Leupeptin, Benzamidine hydrochloride, and Phenylmethylsulfonyl fluoride). Following centrifugation at 12,000 rpm for 10 min at 4 °C, the supernatants were incubated with Ni^2+^-NTA beads for 2 h at 4 °C. The beads were then washed with a wash buffer containing 50 mM Tris–Cl (pH 8), 300 mM NaCl, and 30 mM imidazole (pH 8). Proteins were subsequently eluted using an elution buffer comprising 50 mM Tris (pH 8), 20 mM NaCl, 350 mM imidazole (pH 8), and 5% glycerol. Afterward, dialysis was performed for 4 h in HEKG5 buffer (20 mM Hepes, 1 mM EGTA, 50 mM KCl, and 5% glycerol). However, for SPR experiments, proteins were dialyzed against HBS-N buffer (Hepes-0.01 M, NaCl-0.15 M pH-7.4). Protein purification steps were performed entirely on ice or maintained at 4 °C throughout. The purification of the capping protein CapZ was performed following the previously published protocol (65).

### Actin purification and labeling

Following previously published protocol, 1 g of rabbit muscle acetone powder was thawed and dissolved in 20 ml of G-buffer (10 mM Tris, pH 8, 0.2 mM CaCl_2_, 0.2 mM ATP, 0.5 mM DTT, and 0.1 mM NaN_3_) (66). The mixture was gently rotated using a ROTOSPIN Rotary Mixer at 4 °C for 45 min to ensure thorough mixing and swelling. The solution was then centrifuged at 2000 rpm for 10 min. After centrifugation, the supernatant was collected, while the insoluble pellet was resuspended in fresh 20 ml G-buffer and subjected to the same mixing and low-speed centrifugation steps. The resulting supernatants from both centrifugation steps were combined and filtered through filter paper to remove any remaining insoluble material. We added 2 mM MgCl_2_ and 50 mM KCl to the supernatant solution to initiate actin polymerization for overnight. Next day solid KCl was added to the solution to achieve a final concentration of 800 mM, followed by continuous mixing in magnetic stirrer for 1 h. The solution was then ultracentrifuged at 65,000 rpm for 2 h using a Ti70 rotor. The pellet fraction containing F-actin was resuspended in 12 ml of G-buffer using a dounce homogenizer and then dialyzed against 2 L of G-buffer for 2 days at 4 °C, with a buffer change after a 24 h interval. The dialyzed actin was subjected to ultracentrifugation at 80,000 rpm for 60 min using a TLA-110 rotor. The resulting supernatant was then loaded onto a HiPrep 16/60 Sephacryl S-200 HR (Cytiva) for gel filtration. The collected column fractions were stored at 4 °C.

For the pyrene-actin polymerization assay, actin was fluorescently labeled at cysteine 374 using pyrenyl-iodoacetamide, following the published protocol (67, 68).

### Pyrene-actin polymerization assay

Pyrene-actin polymerization assays were performed using a fluorescence spectrophotometer (QM40, Photon Technology International) (69). A mixture of 10% pyrene-labeled actin and 90% unlabeled monomeric actin was prepared in G-buffer. To initiate the assay, 2 μM of this pyrene-actin mixture was converted to Mg^2+^-actin in exchange buffer (10 mM EGTA and 1 mM MgCl_2_). FMN1 alone, FNBP4 alone, or a preincubated FMN1-FNBP4 mixture was then added to the actin solution. The control group consisted of actin with the control buffer, serving as the actin control. To initiate actin polymerization, 3 μl of a 20X initiation mix was added to achieve a final reaction volume of 60 μl. Fluorescence measurements were recorded over time at 25 °C, with excitation at 365 nm and emission at 407 nm, corresponding to the pyrene fluorescence. Actin polymerization rates were measured from the slopes of the curves between 200 and 300 s and the IC50 value was calculated as the concentration at which the polymerization rate was inhibited by 50%.

### Actin elongation assay

First, 10 μM F-actin was prepared, and 5 μl of it was transferred into a fresh tube containing 30 μl of F-buffer and 15 μl of HEKG5 buffer, with or without proteins. The mixture was mechanically sheared by passing it through a 27-gauge needle five times to generate actin seeds. Subsequently, in another fresh tube, 20 μl of the sheared actin seeds was combined with 0.5 μM G-actin (comprising 10% pyrene-labeled actin and 90% unlabeled actin), G-buffer, and an initiation mix, bringing the total volume to 60 μl. Here, actin seeds were preincubated with CapZ first, followed by the addition of either FH1-FH2 FMN1 alone or FH1-FH2 FMN1 preincubated with WW1-WW2 FNBP4 for this assay. The reaction was then monitored as described previously. Here, the actin seed concentration was 333 nM (61, 70). The actin elongation rates were plotted using the raw slope values measured between 50 and 100 s.

### Cosedimentation assay

G-actin was incubated with F-buffer (10 mM Tris pH 7.5, 0.2 mM DTT, 0.5 mM ATP, 50 mM KCl, 2 mM MgCl_2_, and 0.2 mM CaCl_2_) at 25 °C for 2 h to prepare the initial 25 μM F-actin stock. Subsequently, reactions were set up in ultracentrifuge tubes with a final volume of 50 μl. For cosedimentation assay, we kept the actin concentration at 5 μM in the final reaction volume. Various concentrations of WW1-WW2 FNBP4 proteins were added with F-actin in F-buffer. The samples were then incubated at 25 °C for 10 min. F-actin without FNBP4 served as the actin control, while samples containing only FNBP4 were used as negative controls to confirm protein solubility. The supernatant and pellet fractions were separated by high-speed centrifugation at 90,000 rpm for 30 min at 4 °C using a TLA-100 rotor. The pellet fractions were resuspended in 50 μl of the F-buffer. Equal volumes of the supernatant and pellet samples were then analyzed by SDS-PAGE.

### Bundling assay

F-actin was polymerized by incubating G-actin in F-buffer for 3 h. Subsequently, FNBP4 was incubated with either FH1-FH2 FMN1 or FH2 FMN1 for 10 min. After this incubation, 5 μM of F-actin was added to the reaction mixture and incubated for 1 h. The reaction was then centrifuged at 14,000 rpm for 10 min at 4 °C. The supernatants were collected, while the pellets were resuspended in F-buffer, concentrated fivefold using F-buffer aid quantification. Both the supernatant and pellet fractions were mixed with sample loading buffer and loaded onto a 10% SDS-PAGE gel. Pellet fraction band intensities were measured, with FH1-FH2 FMN1 or FH2 FMN1 serving as a positive control, representing 100% bundle activity. Bundle activity was plotted relative to increasing concentrations of FNBP4, with FNBP4 concentration on the X-axis and bundle activity on the Y-axis.

### TIRF microscopy: Actin nucleation and actin bundling

Glass coverslips (22 × 22 mm) were cleaned using a 2% Hellmanex III solution and subjected to bath sonication at 60 °C for 60 min. Coverslips were then thoroughly rinsed with water and sonicated in 100% ethanol for 1 h. Afterward, the coverslips were rinsed again with water and dried with an N_2_ stream to prevent watermarks on the surface. Coverslips were coated with a poly-L-lysine solution for 45 min, followed by washing with water and air drying. Flow chambers were then assembled by placing double-sided tape on a glass slide and positioning the poly-L-lysine-coated coverslips with the coated side facing inward. Monomeric actin (2 μM) was first converted to Mg^2+^-actin in exchange buffer for 3 min. Subsequently, the actin was mixed with either the preincubated FNBP4-FMN1 mixture, FNBP4 alone, or FMN1 alone in KMEI buffer (50 mM KCl, 1 mM EGTA, 1 mM MgCl_2_, and 10 mM imidazole). Rhodamine–phalloidin was then added, and the mixture was left for 30 s. Finally, the entire reaction was diluted in TIRF buffer (10 mM imidazole pH 7.4, 50 mM KCl, 1 mM MgCl_2_, 1 mM EGTA, 0.2 mM ATP, 10 mM DTT, 1% methylcellulose, 10 μg/ml glucose oxidase, 20 μg/ml catalase, and 15 mM glucose) for visualization. TIRF microscopy was performed using a Nikon Eclipse Ti2 inverted microscope equipped with an Apo TIRF 100x oil immersion objective, two solid-state lasers, and a cooled, back-illuminated ORCA-Flash4.0 Hamamatsu digital camera. The experiments were conducted at room temperature, with the perfect focus system enabled throughout live imaging. Images were captured at 10 s intervals over a period of 10 min.

The TIRF bundling assay was performed following a previously described low-speed centrifugation protocol with slight modifications. Briefly, 2 μM F-actin was incubated with either FH1-FH2 FMN1 or FH2 FMN1 in the presence or absence of WW1-WW2 FNBP4. After incubation, the samples were stained with rhodamine–phalloidin for 1 min. The reaction was then immediately diluted 8-fold with TIRF buffer and visualized using a TIRF microscope.

### Molecular docking and simulation

#### For full-length FMN1 monomer and full-length FNBP4

The 3D structures of full-length FNBP4 (UniProt ID: Q8N3X1) and full-length FMN1 (UniProt ID: Q05860) were predicted using AlphaFold Colab (71), and based on predicted local distance difference test (pLDDT) and predicted template modeling (pTM) scores, the best models were selected for docking. Protein-protein docking was performed using the HADDOCK server (https://rascar.science.uu.nl/haddock2.4/) (72). Initially, we submitted the Protein Data Bank files of the full-length monomeric FMN1 and full-length FNBP4, selecting a maximum of 150 amino acids (the server’s limit for defining interaction surfaces) as the interaction surface before initiating the docking process. For FMN1, we chose 870 to 1020 amino acid stretch encompassing the FH1 domain, the interdomain connector, and part of the FH2 domain. For FNBP4, we selected a stretch of amino acids (214–364) containing the WW1 domain and beyond. The model with the best score from both docking experiments was selected for MD simulations using GROMACS (v2021) (https://www.gromacs.org/) (73). The OPLS (Optimized Potential for Liquid Simulations) force field was used to evaluate interactions and stability, with the system solvated using the SPC216 water model and neutralized using ions added *via* the genion tool. Energy minimization was performed to relax the system, followed by a 10 ns simulation under periodic boundary conditions at a constant temperature of 300 K.

To monitor the stability of the FNBP4-FMN1 complex during the simulation, the RMSD relative to the minimized and equilibrated structure was calculated using GROMACS built-in tools. The Rg was also calculated to evaluate the compactness of the protein complex. Both RMSD and Rg were plotted using GraphPad Prism (v9.5). The interacting residues at the FNBP4-FMN1 interface were identified using PyMOL (v2.5) (https://pymol.org/). LigPlot+ (v2.2) (https://www.ebi.ac.uk/thornton-srv/software/LigPlus/) was used to generate 2D interaction diagrams of FNBP4 and FMN1 complex, highlighting the types of interactions involved (*e.g.*, hydrogen bonds, hydrophobic interactions, and ionic contacts) (74). The dynamic behavior of the protein complex was visualized through trajectory analysis and movie generation using PyMOL.

#### For dimer of Extended FH2 FMN1 and WW1-WW2 FNBP4

As we know, the FH2 domain of formins exists in a dimeric form. To generate the dimeric form of FMN1, we used COSMIC^2^ AlphaFold Multimer (75). Next, we attempted to dock the full-length FMN1 dimer with FNBP4 using HADDOCK. However, we did not find any specific interaction involving the FH1 domain of FMN1 and the WW1 domain of FNBP4. This could be due to HADDOCK's approach, which relies on randomizing orientations and performing rigid-body minimization, treating both the FMN1 dimer and FNBP4 as rigid bodies, thereby limiting conformational flexibility during docking.

Given that we already had biochemical data and MD simulation data, we then focused on docking the Extended FH2 FMN1 dimer with the WW1-WW2 FNBP4. To do this, we first generated 3D structures for both the Extended FH2 FMN1 dimer and the WW1-WW2 FNBP4 using AlphaFold Colab. We performed docking using HADDOCK, where we manually specified the interaction interfaces based on our MD simulation data. We provided the amino acid stretch (961–983) from FMN1, which spans the interdomain connector region, and the WW1 interface (214–248) from WW1-WW2 FNBP4. Due to the limitations of the HADDOCK server, which can only process interactions between two molecules at a time, the initially docked structure of the Extended FH2 FMN1 dimer with one molecule of WW1-WW2 FNBP4 was submitted again to the HADDOCK server for further docking with another molecules of WW1-WW2 FNBP4. For this docking run, we selected an amino acid stretch (residues 961–983) from another part of the FH1-FH2 FMN1 dimer that had not been involved in any interactions with the first WW1-WW2 FNBP4 molecule. Following a similar methodology as discussed previously, we used the docked structure with the best HADDOCK score and proceeded with simulations using the GROMACS software package. RMSD and Rg were plotted using GraphPad Prism. Finally, a trajectory movie for the complex was created in PyMOL.

### Prediction of secondary structure of WW1-WW2 FNBP4

We predicted the 3D structure of the WW1-WW2 FNBP4 using AlphaFold. To validate the secondary structural elements, we conducted CD spectroscopy on purified WW1-WW2 FNBP4. CD spectra were recorded using a Jasco J-815 CD spectrophotometer under constant nitrogen flush. A 0.1 mg/ml solution of WW1-WW2 FNBP4 was prepared in CD buffer (10 mM potassium phosphate, 100 mM potassium chloride) and scanned over a wavelength range of 190 to 300 nm with a scanning speed of 100 nm/min (69). The resulting data were expressed as a mean residue ellipticity (MRE, degree cm^2^/dmol residue) *versus* wavelength curve. Additionally, to assess the structural conservation of the WW1 and WW2 domains, we performed structural superimposition of both domains with nine known WW domains using PyMOL (Supporting Information Table S1).

### Surface plasmon resonance

Binding kinetics of the WW1-WW2 FNBP4 and the extended-FH2 FMN1 constructs were analyzed using a Biacore T200 system (GE HealthCare Life Sciences). WW1-WW2 FNBP4 protein was immobilized on a CM5 sensor chip (Series S) through amine coupling method. For immobilization, a 25 μg/ml solution of WW1-WW2 FNBP4 was prepared in sodium acetate buffer (pH 4.5), and HBS-EP buffer (Hepes 0.01 M, EDTA 0.03 M, NaCl 0.15 M, surfactant P20 0.05%, pH 7.4) was used as the running buffer at a flow rate of 30 μl/min. The immobilization process resulted in a response unit (RU) of 1016.5, with a reference cell used for blank correction. Various concentrations of the Extended FH2 FMN1 protein were then flowed over the WW1-WW2 FNBP4-immobilized surface. HBS-N buffer was used as the running buffer, and the surface was regenerated after each cycle using 10 mM glycine (pH 2.5). The association and dissociation phases were monitored for 120 s and 380 s, respectively. Each concentration was tested in duplicate as a positive control, while a zero-concentration buffer served as a negative control to rule out nonspecific binding. All experiments were conducted at 25 °C, with the sample compartment maintained at 18 °C. The resulting sensograms were analyzed using Biacore Evaluation Software (version 2.0). The data were globally fitted to a 1:1 Langmuir binding model to calculate equilibrium dissociation constants (K_D_), association rates (k_a_), and dissociation rates (k_d_).

### Cells and transfection

HeLa cells (Catalog number CCL-2, Lot number 70046455) were purchased from American Type Culture Collection. Cells were maintained in minimum essential medium and supplemented with 2 mM L-glutamine, 1% penicillin/streptomycin, and 10% fetal bovine serum. HeLa cells were transfected with different GFP constructs of FNBP4 using Lipofectamine-2000 and cultured for 4 h. Afterward, the cells were maintained in fresh media for 12 h and then fixed.

### Antibodies

Anti-FNBP4 sera were raised against the WW1-WW2 FNBP4 construct by immunizing BALB/c mice, as previously described (52). The immunization protocol, spanning 70 days, followed standard procedures and was approved by the Institutional Animal Ethics Committee (IAEC) under protocol reference number IISERK/IAEC/2022/024. Terminal bleeds were collected and validated through Western blot analysis against the recombinant protein and cell lysate.

### Immunofluorescence staining

HeLa cells were seeded on coverslips at a density of 5 × 10^4^ cells/ml and allowed to adhere. After attachment, the cells were fixed and permeabilized with ice-cold acetone and methanol (1:1) solution for 15 min at 4 °C. The permeabilized cells were blocked with 2% bovine serum albumin in PBS for 90 min at room temperature. After blocking, the cells were incubated with primary antibody, diluted in 1% bovine serum albumin-PBST (PBS + 0.075% v/v Tween-20) solution, for 2 h at room temperature. Following primary antibody incubation, the cells were incubated with secondary antibody for 1 h at room temperature. DNA was stained with 4′,6-diamidino-2-phenylindole (DAPI), incorporated in the fluoroshield mounting solution, and the coverslips were subsequently mounted. After each step, cells were washed with either PBS or PBS-T. For immunostaining, mouse anti-FNBP4 was used as the primary antibody at a 1:500 dilution, and Alexa Fluor 488-conjugated anti-mouse IgG (Invitrogen, A-11017) as the secondary antibody at a 1:1000 dilution. Images were captured using a Leica SP8 confocal microscope system equipped with a 63 × /1.40 N.A oil immersion objective (HCPL APO CS2 63×/1.40 OIL) as previously described (69).

### Statistical analysis

All data were analyzed and plotted using GraphPad Prism version 8.4.2. Statistical significance between the means of two groups was evaluated using an unpaired two-tailed Student’s *t* test, *p* < 0.05 considered statistically significant for (Figs. 4*B* and 5*B*). One-way ANOVA with *post hoc* Tukey’s honestly significant difference test was performed for (Fig. 2*B*).

## Data availability

All datasets that support the results of this study are available from the corresponding author upon reasonable request.

## Supporting information

This article contains supporting information.

## Conflict of interest

The authors declare that they have no conflict of interest with the contents of this article.

## References

[1] Dominguez R., Holmes K.C. (2011). Actin structure and function. Annu. Rev. Biophys..

[2] Pollard T.D., Cooper J.A. (2009). Actin, a central player in cell shape and movement. Science.

[3] Blanchoin L., Boujemaa-Paterski R., Sykes C., Plastino J. (2014). Actin dynamics, architecture, and mechanics in cell motility. Physiol. Rev..

[4] Ulferts S., Lopes M., Miyamoto K., Grosse R. (2024). Nuclear actin dynamics and functions at a glance. J. Cell Sci..

[5] Kristó I., Bajusz I., Bajusz C., Borkúti P., Vilmos P. (2016). Actin, actin-binding proteins, and actin-related proteins in the nucleus. Histochem. Cell Biol..

[6] Wollscheid H.-P., Ulrich H.D. (2023). Chromatin meets the cytoskeleton: the importance of nuclear actin dynamics and associated motors for genome stability. DNA Repair.

[7] Liu C., Zhu R., Mao Y. (2018). Nuclear actin polymerized by mDia2 confines centromere movement during CENP-A loading. iScience.

[8] Rajakylä E.K., Vartiainen M.K. (2014). Rho, nuclear actin, and actin-binding proteins in the regulation of transcription and gene expression. Small GTPases.

[9] Baarlink C., Plessner M., Sherrard A., Morita K., Misu S., Virant D. (2017). A transient pool of nuclear F-actin at mitotic exit controls chromatin organization. Nat. Cell Biol..

[10] Nebl G., Meuer S.C., Samstag Y. (1996). Dephosphorylation of serine 3 regulates nuclear translocation of cofilin. J. Biol. Chem..

[11] Skare P., Kreivi J.-P., Bergström A., Karlsson R. (2003). Profilin I colocalizes with speckles and Cajal bodies: a possible role in pre-mRNA splicing. Exp. Cell Res..

[12] Isogai T., Innocenti M. (2016). New nuclear and perinuclear functions of formins. Biochem. Soc. Trans..

[13] Goode B.L., Eck M.J. (2007). Mechanism and function of formins in the control of actin assembly. Annu. Rev. Biochem..

[14] Watanabe N., Kato T., Fujita A., Ishizaki T., Narumiya S. (1999). Cooperation between mDia1 and ROCK in Rho-induced actin reorganization. Nat. Cell Biol..

[15] Kobielak A., Pasolli H.A., Fuchs E. (2004). Mammalian formin-1 participates in adherens junctions and polymerization of linear actin cables. Nat. Cell Biol..

[16] Habas R., Kato Y., He X. (2001). Wnt/frizzled activation of Rho regulates vertebrate gastrulation and requires a novel formin homology protein Daam1. Cell..

[17] Higgs H.N., Peterson K.J. (2005). Phylogenetic analysis of the formin homology 2 domain. MBoC.

[18] Schönichen A., Geyer M. (2010). Fifteen formins for an actin filament: a molecular view on the regulation of human formins. Biochim. Biophys. Acta.

[19] Miki T., Okawa K., Sekimoto T., Yoneda Y., Watanabe S., Ishizaki T. (2009). mDia2 shuttles between the nucleus and the cytoplasm through the importin-{alpha}/{beta}- and CRM1-mediated nuclear transport mechanism. J. Biol. Chem..

[20] Baarlink C., Wang H., Grosse R. (2013). Nuclear actin network assembly by formins regulates the SRF coactivator MAL. Science.

[21] Ménard I., Gervais F.G., Nicholson D.W., Roy S. (2006). Caspase-3 cleaves the formin-homology-domain-containing protein FHOD1 during apoptosis to generate a C-terminal fragment that is targeted to the nucleolus. Apoptosis.

[22] Westendorf J.J. (2001). The formin/diaphanous-related protein, FHOS, interacts with Rac1 and activates transcription from the serum response element. J. Biol. Chem..

[23] Yamada K., Ono M., Bensaddek D., Lamond A.I., Rocha S. (2013). FMN2 is a novel regulator of the cyclin-dependent kinase inhibitor p21. Cell Cycle.

[24] Belin B.J., Lee T., Mullins R.D. (2015). DNA damage induces nuclear actin filament assembly by Formin-2 and Spire-1/2 that promotes efficient DNA repair. eLife.

[25] Higgs H.N. (2005). Formin proteins: a domain-based approach. Trends Biochem. Sci..

[26] Li F., Higgs H.N. (2003). The mouse Formin mDia1 is a potent actin nucleation factor regulated by autoinhibition. Curr. Biol..

[27] Li F., Higgs H.N. (2005). Dissecting requirements for auto-inhibition of actin nucleation by the formin, mDia1. J. Biol. Chem..

[28] Otomo T., Otomo C., Tomchick D.R., Machius M., Rosen M.K. (2005). Structural basis of Rho GTPase-mediated activation of the formin mDia1. Mol. Cell..

[29] Lammers M., Rose R., Scrima A., Wittinghofer A. (2005). The regulation of mDia1 by autoinhibition and its release by Rho∗GTP. EMBO J..

[30] Takeya R., Taniguchi K., Narumiya S., Sumimoto H. (2008). The mammalian formin FHOD1 is activated through phosphorylation by ROCK and mediates thrombin-induced stress fibre formation in endothelial cells. EMBO J..

[31] Staus D.P., Taylor J.M., Mack C.P. (2011). Enhancement of mDia2 activity by Rho-kinase-dependent phosphorylation of the diaphanous autoregulatory domain. Biochem. J..

[32] Wang J., Neo S.P., Cai M. (2009). Regulation of the yeast formin Bni1p by the actin-regulating kinase Prk1p. Traffic.

[33] Chesarone M., Gould C.J., Moseley J.B., Goode B.L. (2009). Displacement of formins from growing barbed ends by bud14 is critical for actin cable architecture and function. Dev. Cell.

[34] Chesarone-Cataldo M., Guérin C., Yu J.H., Wedlich-Soldner R., Blanchoin L., Goode B.L. (2011). The myosin passenger protein Smy1 controls actin cable structure and dynamics by acting as a formin damper. Dev. Cell.

[35] Graziano B.R., Yu H.-Y.E., Alioto S.L., Eskin J.A., Ydenberg C.A., Waterman D.P. (2014). The F-BAR protein Hof1 tunes formin activity to sculpt actin cables during polarized growth. Mol. Biol. Cell.

[36] Garabedian M.V., Stanishneva-Konovalova T., Lou C., Rands T.J., Pollard L.W., Sokolova O.S. (2018). Integrated control of formin-mediated actin assembly by a stationary inhibitor and a mobile activator. J. Cell Biol..

[37] Rands T.J., Goode B.L. (2021). Bil2 is a novel inhibitor of the yeast formin Bnr1 required for proper actin cable organization and polarized secretion. Front. Cell Dev. Biol..

[38] Mason F.M., Heimsath E.G., Higgs H.N., Soderling S.H. (2011). Bi-modal regulation of a formin by srGAP2. J. Biol. Chem..

[39] Brenig J., de Boor S., Knyphausen P., Kuhlmann N., Wroblowski S., Baldus L. (2015). Structural and Biochemical Basis for the Inhibitory Effect of Liprin-α3 on Mouse Diaphanous 1 (mDia1) Function. J Biol Chem.

[40] Ginosyan A.A., Grintsevich E.E., Reisler E. (2019). Neuronal drebrin A directly interacts with mDia2 formin to inhibit actin assembly. Mol. Biol. Cell.

[41] Srapyan S., Tran D.P., Loo J.A., Grintsevich E.E. (2023). Mapping molecular interaction interface between diaphanous formin-2 and neuron-specific drebrin A. J. Mol. Biol..

[42] Wang H., Hu J., Yi K., Ma Z., Song X., Lee Y. (2022). Dual control of formin-nucleated actin assembly by the chromatin and ER in mouse oocytes. Curr. Biol..

[43] Ramabhadran V., Hatch A.L., Higgs H.N. (2013). Actin monomers activate inverted formin 2 by competing with its autoinhibitory interaction. J. Biol. Chem..

[44] A M., Fung T.S., Kettenbach A.N., Chakrabarti R., Higgs H.N. (2019). A complex containing lysine-acetylated actin inhibits the formin INF2. Nat. Cell Biol..

[45] Zhou F., Leder P., Martin S.S. (2006). Formin-1 protein associates with microtubules through a peptide domain encoded by exon-2. Exp. Cell Res..

[46] Maas R.L., Zeller R., Woychik R.P., Vogt T.F., Leder P. (1990). Disruption of formin-encoding transcripts in two mutant limb deformity alleles. Nature.

[47] Zhou F., Leder P., Zuniga A., Dettenhofer M. (2009). Formin1 disruption confers oligodactylism and alters Bmp signaling. Hum. Mol. Genet..

[48] Chan D.C., Wynshaw-Boris A., Leder P. (1995). Formin isoforms are differentially expressed in the mouse embryo and are required for normal expression of fgf-4 and shh in the limb bud. Development.

[49] Li N., Mruk D.D., Wong C.K.C., Han D., Lee W.M., Cheng C.Y. (2015). Formin 1 regulates ectoplasmic specialization in the rat testis through its actin nucleation and bundling activity. Endocrinology.

[50] Simon-Areces J., Dopazo A., Dettenhofer M., Rodriguez-Tebar A., Garcia-Segura L.M., Arevalo M.-A. (2011). Formin1 mediates the induction of dendritogenesis and synaptogenesis by Neurogenin3 in mouse hippocampal neurons. PLoS One.

[51] Dettenhofer M., Zhou F., Leder P. (2008). Formin 1-isoform IV deficient cells exhibit defects in cell spreading and focal adhesion formation. PLoS One.

[52] Das S., Maiti S. (2024). Probing the ligand binding specificity of FNBP4 WW domains and interaction with FH1 domain of FMN1. Curr. Res. Struct. Biol..

[53] Varadi M., Anyango S., Deshpande M., Nair S., Natassia C., Yordanova G. (2022). AlphaFold protein structure database: massively expanding the structural coverage of protein-sequence space with high-accuracy models. Nucleic Acids Res..

[54] Aspenström P. (2010). Formin-binding proteins: modulators of formin-dependent actin polymerization. Biochim. Biophys. Acta.

[55] Paul A.S., Pollard T.D. (2008). The role of the FH1 domain and profilin in formin-mediated actin-filament elongation and nucleation. Curr. Biol..

[56] Courtemanche N., Henty-Ridilla J.L. (2024). Actin filament dynamics at barbed ends: new structures, new insights. Curr. Opin. Cell Biol..

[57] Otomo T., Tomchick D.R., Otomo C., Panchal S.C., Machius M., Rosen M.K. (2005). Structural basis of actin filament nucleation and processive capping by a formin homology 2 domain. Nature.

[58] Yamashita M., Higashi T., Suetsugu S., Sato Y., Ikeda T., Shirakawa R. (2007). Crystal structure of human DAAM1 formin homology 2 domain. Genes Cells.

[59] Lu J., Meng W., Poy F., Maiti S., Goode B.L., Eck M.J. (2007). Structure of the FH2 domain of Daam1: implications for formin regulation of actin assembly. J. Mol. Biol..

[60] Chan D.C., Leder P. (1996). Genetic evidence that formins function within the nucleus. J. Biol. Chem..

[61] Breitsprecher D., Jaiswal R., Bombardier J.P., Gould C.J., Gelles J., Goode B.L. (2012). Rocket launcher mechanism of collaborative actin assembly defined by single-molecule imaging. Science.

[62] Zheng K.-W., Zhang C.-H., Wu W., Zhu Z., Gong J.-P., Li C.-M. (2023). FNBP4 is a potential biomarker associated with cuproptosis and promotes tumor progression in hepatocellular carcinoma. Int. J. Gen. Med..

[63] Lawson C.D., Peel S., Jayo A., Corrigan A., Iyer P., Baxter Dalrymple M. (2022). Nuclear fascin regulates cancer cell survival. Elife.

[64] Isogai T., van der Kammen R., Goerdayal S.S., Heck A.J.R., Altelaar A.F.M., Innocenti M. (2015). Proteomic analyses uncover a new function and mode of action for mouse homolog of Diaphanous 2 (mDia2). Mol. Cell Proteomics.

[65] Dutta P., Das S., Maiti S. (2017). Non diaphanous formin delphilin acts as a barbed end capping protein. Exp. Cell Res..

[66] Pollard T.D. (1984). Polymerization of ADP-actin. J. Cell Biol..

[67] Doolittle L.K., Rosen M.K., Padrick S.B. (2013). Measurement and analysis of in vitro actin polymerization. Methods Mol. Biol..

[68] Kouyama T., Mihashi K. (1981). Fluorimetry study of N-(1-pyrenyl)iodoacetamide-labelled F-actin. Local structural change of actin protomer both on polymerization and on binding of heavy meromyosin. Eur. J. Biochem..

[69] Das S., Das S., Rath P.P., Banerjee A., Gourinath S., Mukhopadhyay A.K. (2024). Hemolysin coregulated protein (HCP) from Vibrio cholerae interacts with the host cell actin cytoskeleton. ACS Infect. Dis..

[70] Schönichen A., Mannherz H.G., Behrmann E., Mazur A.J., Kühn S., Silván U. (2013). FHOD1 is a combined actin filament capping and bundling factor that selectively associates with actin arcs and stress fibers. J. Cell Sci..

[71] Mirdita M., Schütze K., Moriwaki Y., Heo L., Ovchinnikov S., Steinegger M. (2022). ColabFold: making protein folding accessible to all. Nat. Methods.

[72] van Zundert G.C.P., Rodrigues J.P.G.L.M., Trellet M., Schmitz C., Kastritis P.L., Karaca E. (2016). The HADDOCK2.2 web server: user-friendly integrative modeling of biomolecular complexes. J. Mol. Biol..

[73] Lemkul J.A. (2024). Introductory tutorials for simulating protein dynamics with GROMACS. J. Phys. Chem. B.

[74] Wallace A.C., Laskowski R.A., Thornton J.M. (1995). LIGPLOT: a program to generate schematic diagrams of protein-ligand interactions. Protein Eng..

[75] Cianfrocco M.A., Wong-Barnum M., Youn C., Wagner R., Leschziner A. (2017). Practice and Experience in Advanced Research Computing 2017: Sustainability, Success and Impact.

