# Supplementary material for: Nuclear protein FNBP4: A novel inhibitor of non-diaphanous formin FMN1-mediated actin cytoskeleton dynamics
Source: J Biol Chem. 2025 Apr 30;301(6):108550. doi: 10.1016/j.jbc.2025.108550 (PMC12159680; doi:10.1016/j.jbc.2025.108550)
Supplement: Supporting Information [file mmc1.docx]

Supporting Information for

**Nuclear Protein FNBP4: A Novel Inhibitor of Non-diaphanous Formin FMN1-Mediated Actin Cytoskeleton Dynamics**

Shubham Das, Saikat Das, Amrita Maity, Sankar Maiti*

Department of Biological Sciences, Indian Institute of Science Education and Research Kolkata, Mohanpur, West Bengal, India.

*Address correspondence to:

Sankar Maiti, Ph.D., Department of Biological Sciences, Indian Institute of Science Education and Research Kolkata, Mohanpur, Nadia - 741246, West Bengal, India.

E-mail: spm@iiserkol.ac.in

**This PDF file includes**

Table S1 to S3

Figure S1 to S10

Video Legends

**Table S1: RMSD analysis table comparing FNBP4 WW1 and WW2 domains with the corresponding WW1 and WW2 domains of PIN1, YAP1, Nedd 4-2, FBP11, GAS-7, APBB-3, PROTOTYPE, WBP4, and FBP28.**

| **WW containing proteins** | **RMSD values (A°) with respect to FNBP4 WW1** | **RMSD values (A°) with respect to FNBP4 WW2** |
| --- | --- | --- |
| **PIN 1 (PDB ID: 2M8I)** | **0.695** | **1.009** |
| **YAP 1 (PDB ID: 5YDY)** | **1.264** | **1.784** |
| **Nedd4-2 (PDB ID: 1WR4)** | **0.569** | **0.820** |
| **FBP 11 (PDB ID: 1ZR7)** | **0.665** | **0.461** |
| **GAS-7 (PDB ID: 2YSH)** | **0.760** | **1.609** |
| **APBB 3 (PDB ID: 2YSC)** | **0.750** | **2.986** |
| **PROTOTYPE (PDB ID: 1E0M)** | **0.951** | **1.801** |
| **WBP4 (PDB ID: 2DK1)** | **0.665** | **0.567** |
| **FBP 28 (PDB ID: 1E0L)** | **1.617** | **1.860** |

**Table S2: Detailed list of oligonucleotides used for cloning FMN1 and FNBP4 constructs.**

| **Name of constructs** | **Vector** | **Primer sequences** | **Source** |
| --- | --- | --- | --- |
| N-terminal WW1-WW2  FNBP4 (214-629 aa) | pET28a+ | FWD 5'-GGCGGATCCGGAATTGAGATGGGCGATTG-3'  REV 5'-GGACTCGAG**TTA**ACCATCTGGAAACTCCCACTG-3' | (1) |
| N-terminal ∆WW1 FNBP4 (249-629 aa) | pET28a+ | FWD 5’-GGCCGGATCCCTTGCCACACAGGTACAGGG-3’  REV 5'-GGACTCGAG**TTA**ACCATCTGGAAACTCCCACTG-3' | (1) |
| FL FNBP4  (1-1017 aa) | pEGFP-C1 | FWD 5’-GGACTCGAGATGGGGAAGAAGTCCCGG-3'  REV 5’-GGCGGATCC**TTA**TGTGTTTGGAGCCATTTTCCTTCT-3’ | This paper |
| NLS1 FNBP4  (1-1004 aa) | pEGFP-C1 | FWD 5’-GGACTCGAGATGGGGAAGAAGTCCCGG-3'  REV 5’-GGCGGATCC**TTA**CCAATCCTCAGGAAGGGCTTC-3’ | This paper |
| N-ter FNBP4  (1-629 aa) | pEGFP-C1 | FWD 5’-GGACTCGAGATGGGGAAGAAGTCCCGG-3'  REV 5’-GGCGGATCC**TTA**ACCATCTGGAAACTCCCACTG-3’ | This paper |
| NLS2 FNBP4  (801-1017 aa) | pEGFP-C1 | FWD 5’- GGACTCGAGGTTCAGAGGTCAGCTACCATTGGC-3’  REV 5’-GGCGGATCC**TTA**TGTGTTTGGAGCCATTTTCCTTCT-3’ | This paper |
| Δ NLS FNBP4  (801-1004 aa) | pEGFP-C1 | FWD 5’- GGACTCGAGGTTCAGAGGTCAGCTACCATTGGC-3’  REV 5’-GGCGGATCC**TTA**CCAATCCTCAGGAAGGGCTTC-3’ | This paper |
| C-terminal FH1-FH2 FMN1 (870-1466 aa) | pET28a+ | FWD 5'-GGCGGATCCCCTCCAGCTCCCCCCACG-3'  REV 5'-GGACTCGAG**TTA**GTTGGTGGCCACGCTGGCT-3' | (1) |
| C-terminal FH2 FMN1 (983-1466 aa) | pET28a+ | FWD 5'-GGCGGATCCCGTAAACCAGCCATTGAGCCC-3'  REV 5'-GGACTCGAG**TTA**GTTGGTGGCCACGCTGGCT-3' | (1) |
| C-terminal FH1 FMN1 (870-970 aa) | pET28a+ | FWD 5'-GGCGGATCCCCTCCAGCTCCCCCCACG-3'  REV 5’-GGACTCGAG**TTA**CAGTCCAGGAGGAGGTGGGGG-3’ | (1) |
| Extended FH2 FMN1  (861-1466 aa) | pET28a+ | FWD 5’- GGCGGATCCGGACTTGCACCCCCACCTCC -3’  REV 5'-GGACTCGAG**TTA**GTTGGTGGCCACGCTGGCT-3' | This paper |

**Table S3: Detailed list of reagents and tools.**

| **Reagent and/or Resources** | **Reference or Source** | **Identifier or Catalog Number** |
| --- | --- | --- |
| Antibodies | | |
| Mouse Anti-FNBP4 | This paper | N/A |
| Alexa Fluor™ 488-conjugated  anti-mouse IgG | Invitrogen | A-11017 |
| Bacterial competent cells and Mammalian cells | | |
| XL10 Gold | Stratagene, Agilent Technologies | 200314 |
| BL21(DE3) | Agilent Technologies | 200131 |
| HeLa cell | ATCC | CCL-2 |
| Recombinant DNA | | |
| pET28+ | Novagen, Merck Life Science | 69864 |
| pEGFP-C1 | Clontech Laboratories, Takara Bio | 6084-1 |
| pEGFPC2-FmnIso1a | Addgene | 19320 |
| Human FNBP4 cDNA | Dharmacon | MHS6278-202758566 |
| CapZ (α and β) | (2) | N/A |
| Chemicals, Enzymes and other reagents | | |
| BamHI | NEB | R0136S |
| XhoI | NEB | R0146S |
| T4 Ligase | Thermo | EL0011 |
| IGEPAL | Merck | 56741 |
| EDTA | Sigma Aldrich | E4884 |
| Benzamidine hydrochloride | Sigma Aldrich | B6506 |
| Paraformaldehyde | Sigma Aldrich | 158127 |
| Poly-L-lysine | Sigma Aldrich | P9155 |
| Aprotinin | SRL | 62179 |
| Imidazole | US Biologicals | 452259 |
| DTT | HiMedia | MB070 |
| Pepstatin A | Alfa Aesar | J60237 |
| Leupeptin | Alfa Aesar | J61188 |
| EGTA | VWR | 20308.156 |
| HEPES | Biowest | P5455 |
| Glycerol | Sigma-Aldrich | G7893 |
| Sodium azide | Sigma-Aldrich | S2002 |
| ATP | Abcam | AB156525 |
| MgCl2 | Sigma-Aldrich | M8266 |
| IPTG | HiMedia | RM2578 |
| Ni-NTA Beads | Qiagen | 30210 |
| Calcium chloride | Sigma-Aldrich | C4901 |
| Hellmanex III solution | Merck | Z805939 |
| N-(1-pyrene) iodoactamide | Invitrogen | P-29 |
| Sodium dodecyl sulfate | Sigma-Aldrich | L4509 |
| Surfactant P20 | Cytiva | BR100054 |
| Series S Sensor Chip CM5 | Cytiva | BR100530 |
| Penicillin-Streptomycin | Gibco | 15140122 |
| MEM | Gibco | 61100061 |
| Fetal Bovine Serum | Gibco | 16000044 |
| L-Glutamine | Gibco | 25030081 |
| Lipofectamine-2000 | Invitrogen | 11668500 |
| Acetone | Merck | SI9F690655 |
| Methanol | FINAR | 389302C250 |
| Bovine Serum Albumin | Sigma-Aldrich | A9418 |
| TWEEN 20 | Sigma-Aldrich | P1379 |
| Fluoroshield with DAPI | Sigma-Aldrich | F6057 |
| Protease inhibitor cocktail | Sigma-Aldrich | P8340 |
| Rhodamine Phalloidin | Invitrogen | R415 |
| 10mM Glycine (pH 2.5) | Cytiva | BR100356 |
| Amine Coupling Kit | Cytiva | BR100050 |
| Software | | |
| UniProt | https://www.uniprot.org/ | N/A |
| PyMOL (v2.5) | https://pymol.org/ | N/A |
| HADDOCK server | https://rascar.science.uu.nl/haddock2.4/ | N/A |
| GROMACS (v2021) | https://www.gromacs.org/ | N/A |
| LigPlot+ (v2.2) | https://www.ebi.ac.uk/thornton-srv/software/LigPlus/ | N/A |
| Fiji ImageJ.2 | https://imagej.net/software/fiji/ | N/A |
| Biacore T200 Evaluation  software (v3.1) | Cytiva | N/A |
| LAS X | Leica | N/A |
| GraphPad Prism 8 | https://www.graphpad.com/ | N/A |

**
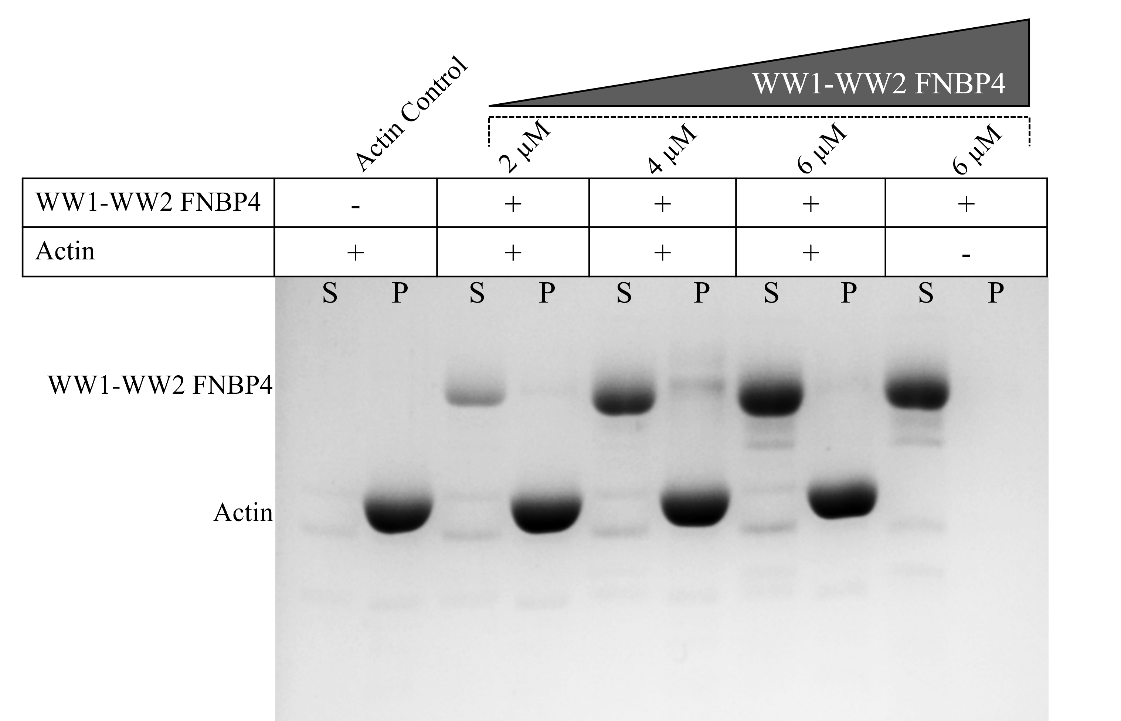
**

**Figure S1. N-ter WW1-WW2 FNBP4 did not interacts with actin.** F-actin co-sedimentation assay: 5 μM F-actin was incubated with or without N-terminal WW1-WW2 FNBP4 for 10 min at room temperature, followed by ultracentrifugation. The supernatant (S) and pelleted (P) fractions were collected and analyzed by Coomassie-stained 10% SDS-PAGE. Proteins that bind to F-actin will co-sediment with the F-actin, appearing in the pellet fraction.


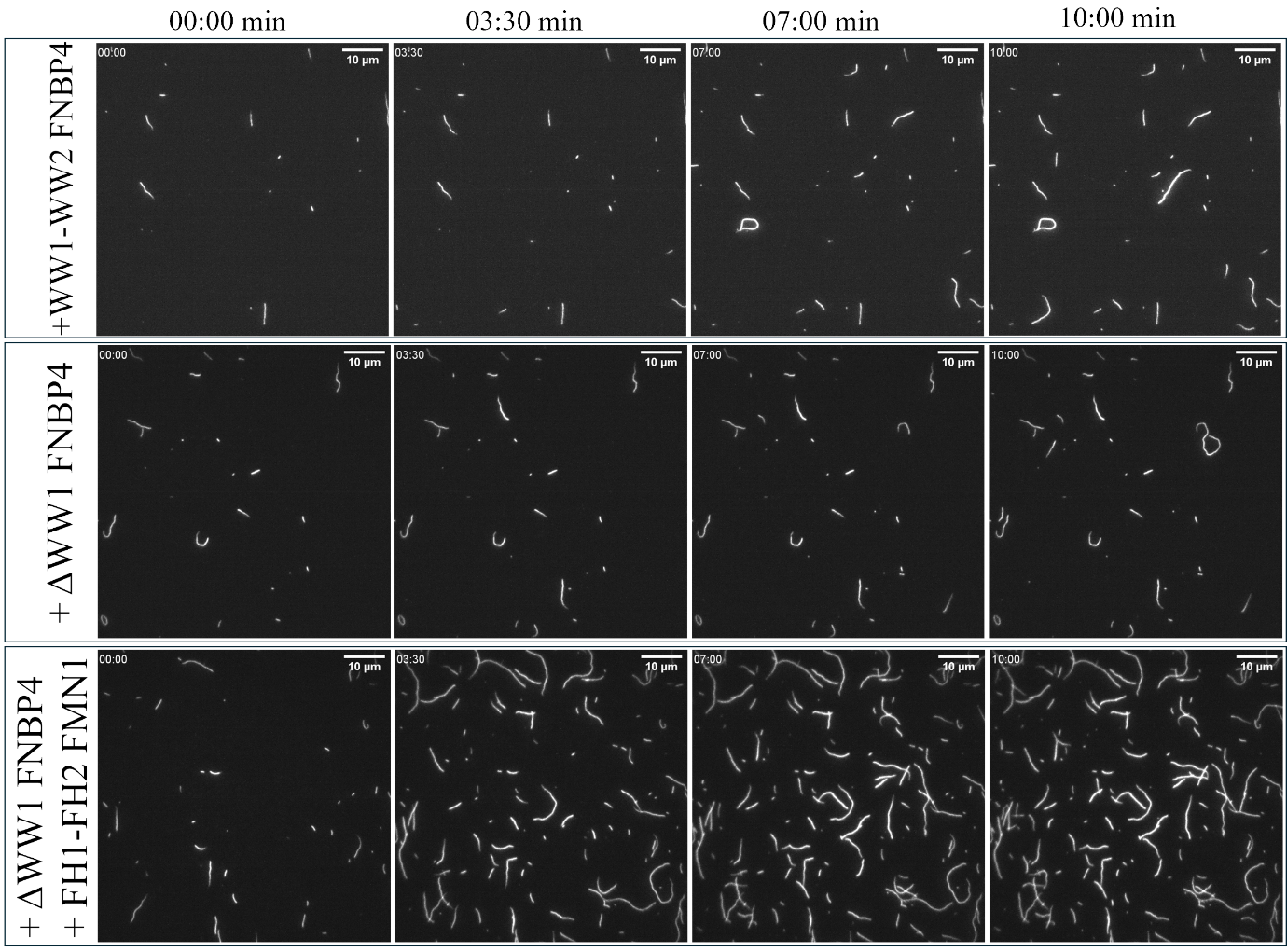


**Figure S2.** **N-terminal ∆WW1 FNBP4 did not inhibit FH1-FH2 FMN1-mediated actin nucleation.** Time-lapse microscopy images of actin filament assembly under the conditions specified in 400 nM N-terminal WW1-WW2 FNBP4, or with 400 nM N-terminal ∆WW1 FNBP4 or with 50 nM FH1-FH2 FMN1 and 400 nM N-terminal ∆WW1 FNBP4. Scale bar is 10 µm.


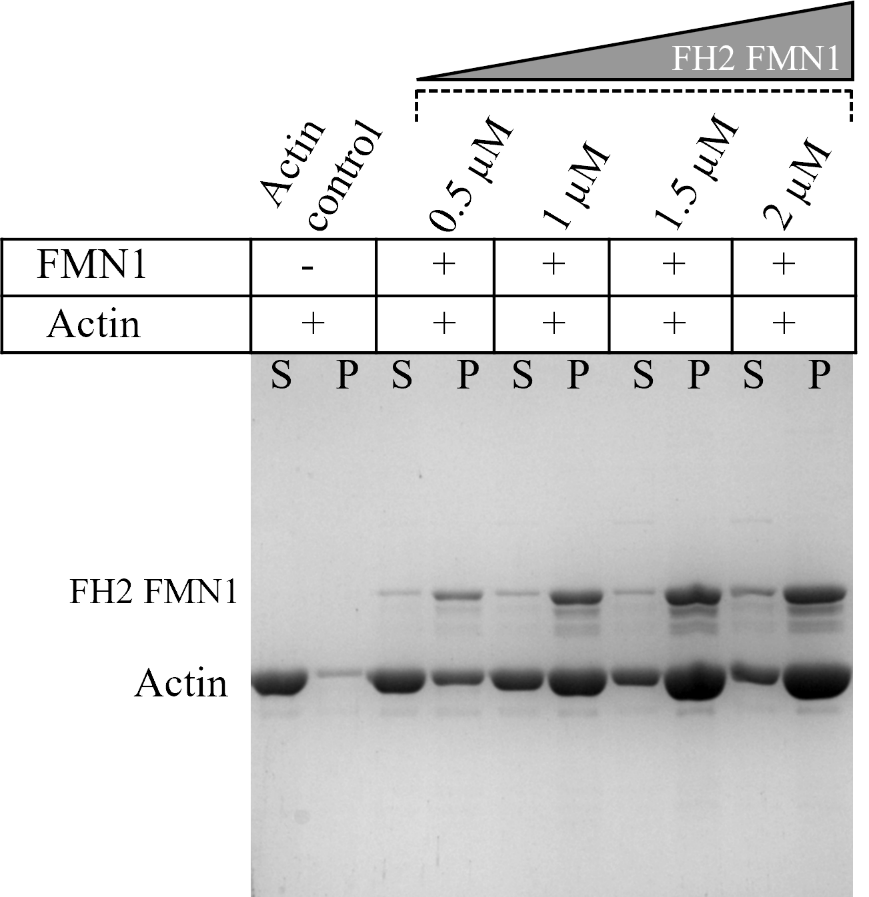


**Figure S3. FMN1 bundle actin filaments.** Low-speed centrifugation assay with different concentrations of FH2 FMN1 incubated with preformed 5 µM F-actin in F-buffer. Co-sedimentation was analyzed by low-speed centrifugation. The supernatant (S) and pelleted (P) fractions were collected and analyzed by Coomassie-stained 10% SDS-PAGE. Pellets were concentrated 5-fold for better visualization.


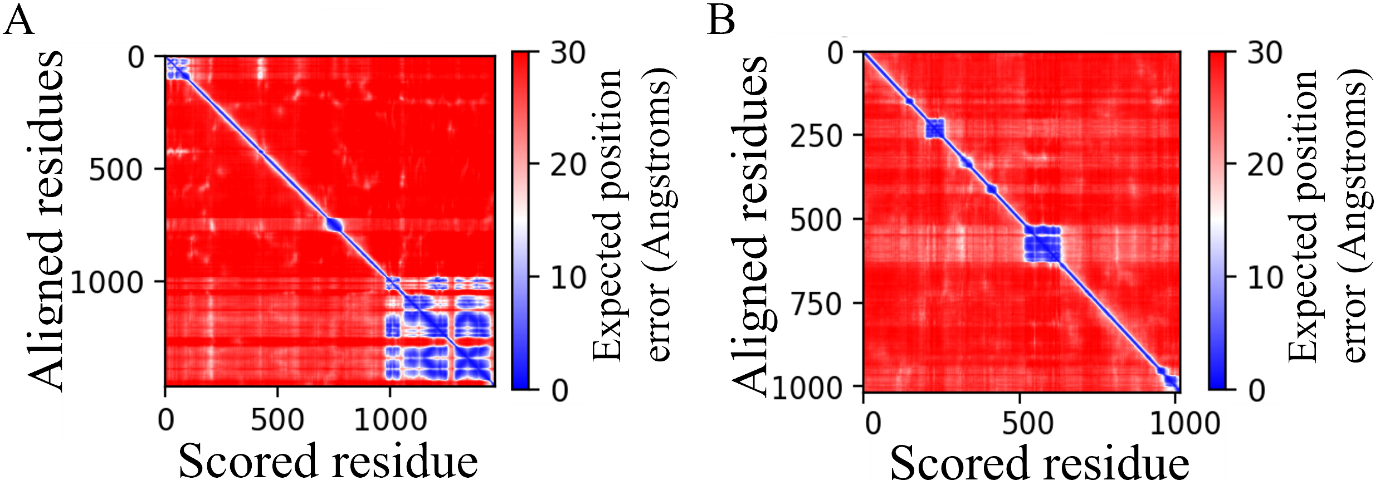


**Figure S4. Structural Prediction of full length FMN1 monomer and full length FNBP4.** Predicted Alignment Error (PAE) heatmap for full length FMN1 monomer (A) and full length FNBP4 (B). The heatmap displays regions of high prediction accuracy in blue tiles and areas with lower confidence in red tiles for the structural models of the respective proteins. (A) For FMN1, the large blue region corresponds to the FH2 domain, indicating high confidence. (B) For FNBP4, two prominent blue regions are observed, corresponding to the WW1 and WW2 domains, reflecting accurate predictions for these domains.

**
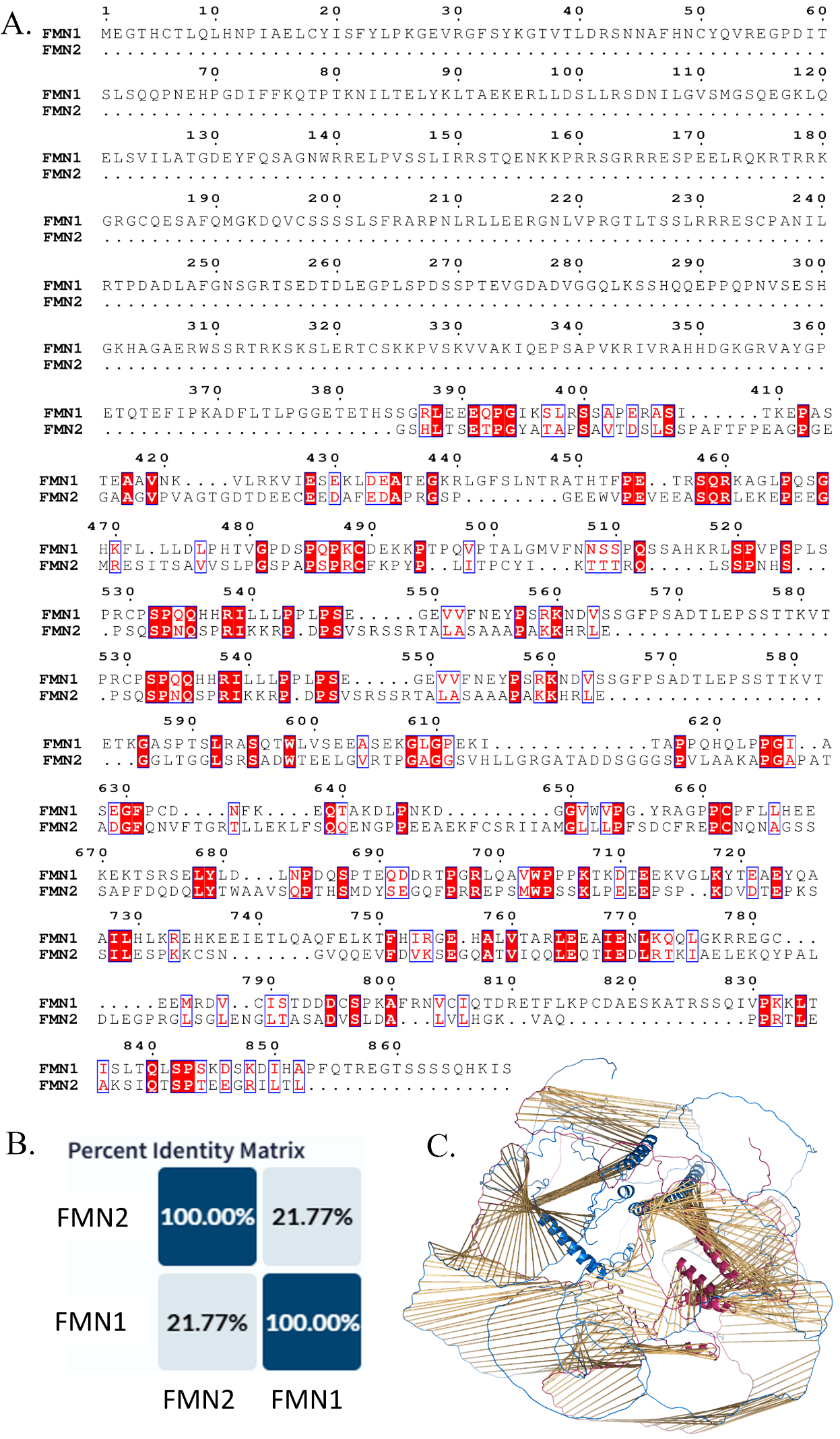
**

**Figure S5. Structural prediction, pairwise sequence alignment, and structural superimposition demonstrate a lack of structural similarity between the SLD domain of FMN2 (residues 274-735) and the N-terminal region of FMN1 (residues 1-869).** (A) Pairwise sequence alignment of the SLD domain of FMN2 (274-735 aa) and the N-terminal of FMN1 (1-869 aa). Completely conserved residues are highlighted with a red background and white letters, while well-conserved residues are indicated by a blue line and red-colored letters. (B) Percent identity matrix of the pairwise sequence alignment between the SLD domain of FMN2 (274-735 aa) and the N-terminal of FMN1 (1-869 aa). (C) Superimposition of the SLD domain of FMN2 (274-735 aa) (shown in marine) and the N-terminal of FMN1 (1-869 aa) (shown in magenta) (RMSD= 53.129 Å).

**
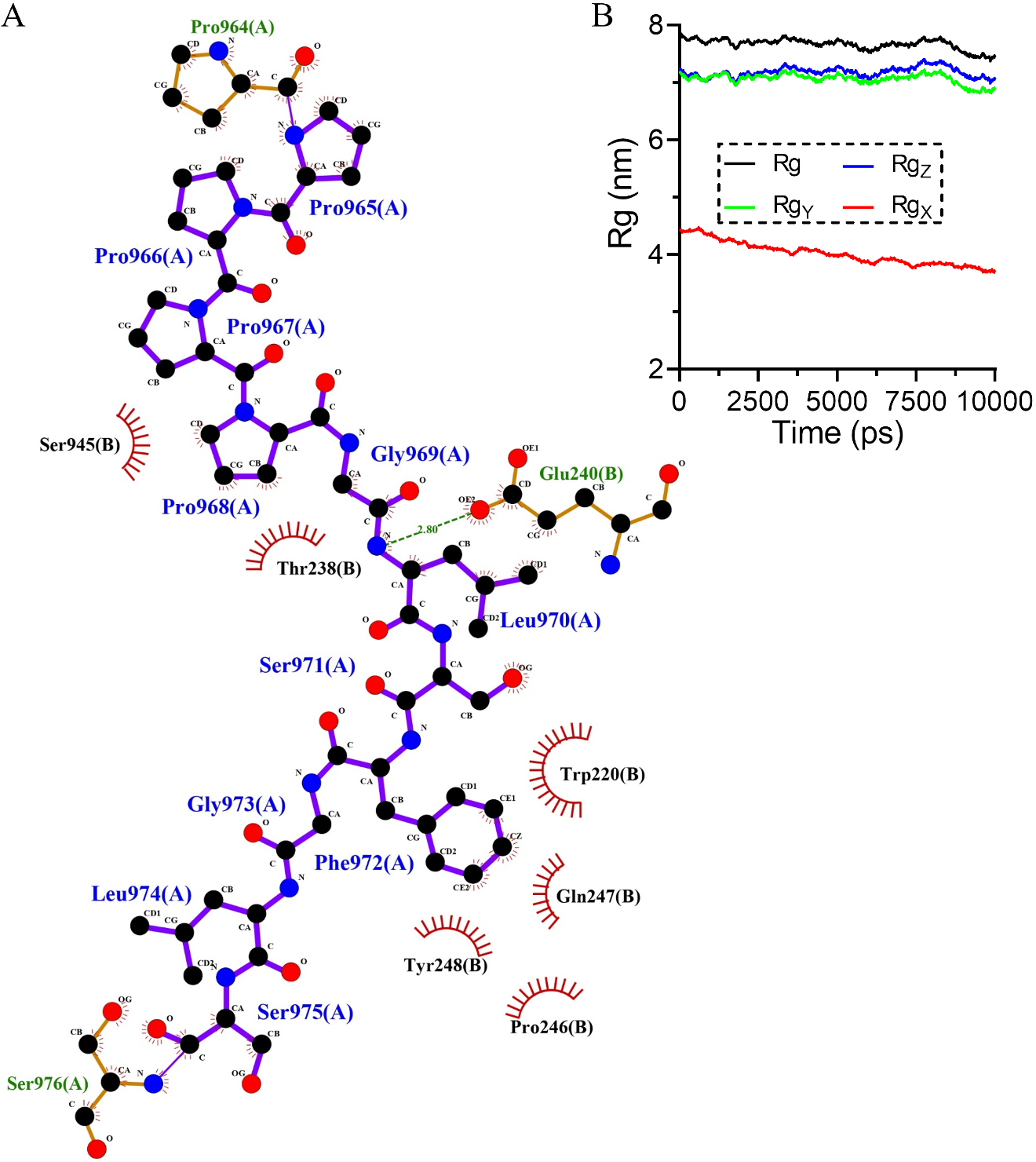
**

**Figure S6. Interaction interface analysis of full length FMN1 monomer-full length FNBP4 complex.** (A) LigPlot+ 2D representation of the protein-protein interaction between FNBP4 and FMN1. The 2D map highlights a hydrophobic interaction network between residues within the WW1 domain of FNBP4 and the interdomain connector region of FMN1. The green dashed line represents a hydrogen bond between Glu240 of FNBP4 and Leu970 of FMN1. Circular spokes represent hydrophobic interactions. (B) The plot illustrates radius of gyration (Rg) (in black) analysis, depicting the compactness of the FMN1-FNBP4 complex during the 10 ns MD simulation. Rg_x_ (in red), Rg_y_ (in green), and Rg_z_ (in blue) represent the radius of gyration of the protein along the x, y, and z axes, respectively.

**
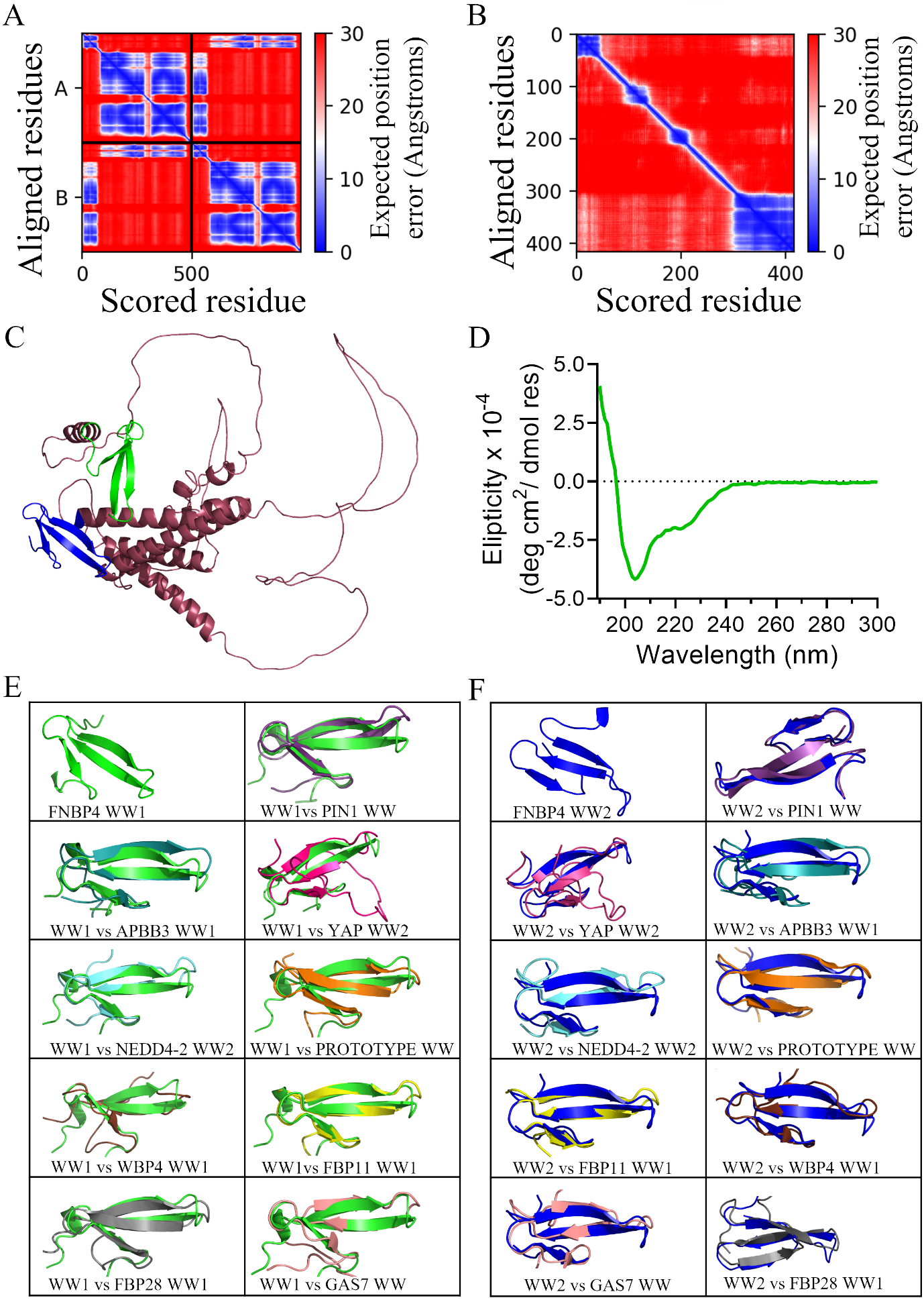
**

**Figure S7. Structural prediction of the extended FH2 FMN1 dimer and the WW1-WW2 FNBP4. Secondary structural analysis of WW1-WW2 FNBP4, WW1 and WW2 domain.** (A) The PAE heatmap shows two large blue tiles in opposite orientations, corresponding to the antiparallel FH2 dimer, indicating high confidence. (B) The two prominent blue tiles at the opposite end of the PAE heatmap for FNBP4 correspond to the WW1 and WW2 domains. (C) Ribbon diagram of WW1-WW2 FNBP4, with WW1 shown in green, WW2 in blue, and the remaining helical and disordered regions in raspberry red. (D) Circular dichroism (CD) spectra of WW1-WW2 FNBP4 in a buffer containing 10 mM potassium phosphate and 100 mM potassium chloride. (E) Structural superimposition of the FNBP4 WW1 domain (green) with WW1 domains from PIN1 (purple), YAP1 (hot pink), Nedd4-2 (aquamarine blue), FBP11 (yellow), GAS-7 (salmon red), APBB-3 (teal blue), PROTOTYPE (orange), WBP4 (chocolate brown), and FBP28 (grey). (F) Superimposition of the FNBP4 WW2 domain (blue) with WW2 domains from PIN1 (purple), YAP1 (hot pink), Nedd 4-2 (aquamarine blue), FBP11 (yellow), GAS-7 (salmon red), APBB-3 (teal blue), PROTOTYPE (orange), WBP4 (chocolate brown), and FBP28 (grey).

**
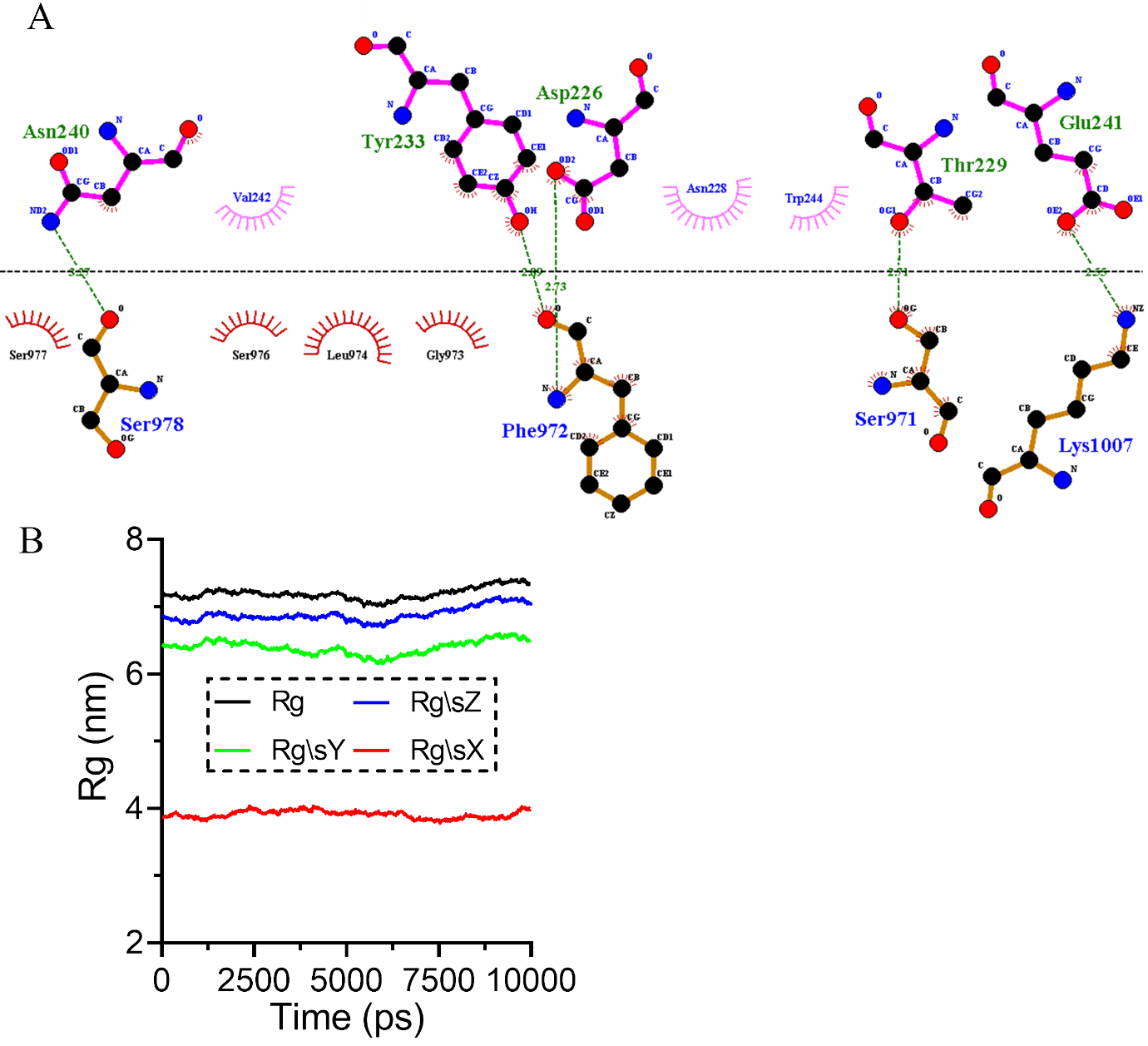
**

**Figure S8. Interaction Interface analysis of the Extended FH2 FMN1 dimer in complex with WW1-WW2 FNBP4.** (A) A 2D interaction map of the Extended FH2-FMN1 dimer with WW1-WW2 FNBP4 complex highlights a hydrophobic interaction network between residues in the WW1 domain of FNBP4 and the interdomain connector region of FMN1. The green dashed line represents a hydrogen bond and Circular spokes represent hydrophobic interactions. (B) The radius of gyration plot (In black line) of the Extended FH2 FMN1 dimer-WW1-WW2 FNBP4 complex reveals the compactness of the complex throughout the 10 ns MD simulation.

**
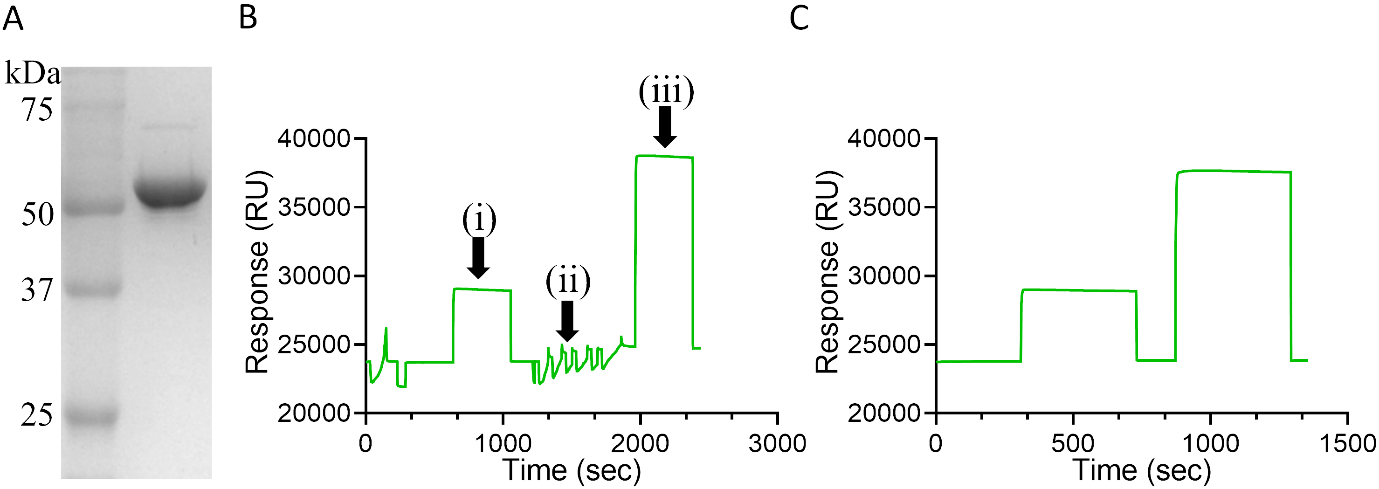
**

**Figure S9. Purification of Extended FH2 FMN1 and immobilization of N-terminal WW1-WW2 FNBP4 for SPR analysis.** (A) Coomassie-stained 10% SDS-PAGE gel showing the protein band of the purified extended FH2 fragment of FMN1. (B) N-terminal WW1-WW2 FNBP4 immobilization on reference channel 4. (i) Activation of the chip surface using EDC/NHS chemistry. (ii) A 25 μg/mL solution of WW1-WW2 FNBP4 was prepared in sodium acetate buffer (pH 4.5) and applied to the surface for immobilization. (iii) Surface deactivation with ethanolamine. (C) Blank immobilization on reference channel 3, without N-terminal WW1-WW2 FNBP4.


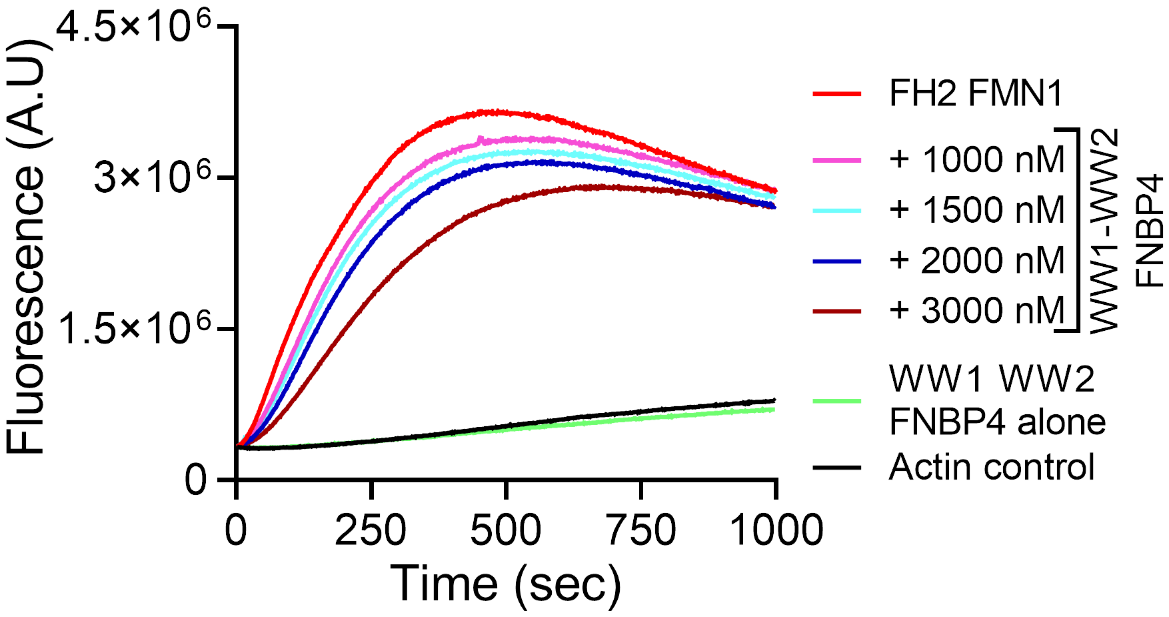


**Figure S10.** Pyrene-actin polymerization assay (2 µM actin monomer, 10% pyrene labeled): Actin polymerization was performed using 50 nM of the FH2 FMN1 construct and/or increasing concentrations of the N-terminal WW1-WW2 FNBP4.

**References:**

1. Das, S., and Maiti, S. (2024) Probing the ligand binding specificity of FNBP4 WW domains and interaction with FH1 domain of FMN1. *Current Research in Structural Biology*. **7**, 100119

2. Dutta, P., Das, S., and Maiti, S. (2017) Non diaphanous formin delphilin acts as a barbed end capping protein. *Experimental Cell Research*. **357**, 163–169

**Video Legends:**

**Video 1:** Time-lapse TIRF imaging of actin filament nucleation in the control assay. This movie shows actin polymerization in the actin-only sample. A total of 61 frames were captured from a 10-minute video, with each frame representing a snapshot taken every 10 seconds.

**Video 2:** Time-lapse TIRF imaging of actin filament nucleation in the presence of 50 nM FH1-FH2 FMN1. This movie demonstrates actin polymerization with 50 nM FH1-FH2 FMN1. A total of 61 frames were captured from a 10-minute video, with frames taken every 10 seconds.

**Video 3:** Time-lapse TIRF imaging of actin filament nucleation with 50 nM FH2 FMN1. The movie shows actin polymerization in the presence of 50 nM FH2 FMN1. A total of 61 frames were captured from a 10-minute video, with each frame recorded every 10 seconds.

**Video 4:** Time-lapse TIRF imaging of actin filament nucleation with 400 nM WW1-WW2 FNBP4. The movie demonstrates that 400 nM WW1-WW2 FNBP4 has no effect on actin polymerization. A total of 61 frames were captured from a 10-minute video, with snapshots taken every 10 seconds.

**Video 5:** Time-lapse TIRF imaging of 50 nM FH1-FH2 FMN1 in the presence of 400 nM WW1-WW2 FNBP4 during actin filament nucleation. This movie shows that 400 nM WW1-WW2 FNBP4 inhibits 50 nM FH1-FH2 FMN1-mediated actin assembly. A total of 61 frames were taken over 10 minutes, with each frame captured every 10 seconds.

**Video 6:** Time-lapse TIRF imaging of 50 nM FH2 FMN1 in the presence of 400 nM WW1-WW2 FNBP4 during actin filament nucleation. The movie indicates that 400 nM WW1-WW2 FNBP4 does not significantly inhibit 50 nM FH2 FMN1-mediated actin polymerization. A total of 61 frames were captured over 10 minutes, with frames taken every 10 seconds.

**Video 7:** Time-lapse TIRF imaging of actin nucleation with 400 nM ΔWW1 FNBP4. The movie demonstrates that 400 nM ΔWW1 FNBP4 has no significant effect on actin polymerization. A total of 61 frames were captured from a 10-minute video, with each frame taken every 10 seconds.

**Video 8:** Time-lapse TIRF imaging of 50 nM FH1-FH2 FMN1 in the presence of 400 nM ΔWW1 FNBP4 during actin filament nucleation. The movie shows no significant effect of 400 nM ΔWW1 FNBP4 on 50 nM FH1-FH2 FMN1-mediated actin polymerization. A total of 61 frames were taken from a 10-minute video, with snapshots every 10 seconds.

**Video 9:** MD simulation of the full-length monomeric FMN1 and full-length FNBP4 complex. This movie illustrates the MD trajectory of the FMN1-FNBP4 complex in a solvated environment over 10 ns. FMN1 is shown in teal blue, while FNBP4 is depicted in raspberry red.

**Video 10:** MD simulation of the Extended FH2 FMN1 dimer in complex with two molecules of WW1-WW2 FNBP4. This movie illustrates the MD trajectory of the Extended FH2 FMN1 dimer in complex with two molecules of WW1-WW2 FNBP4 in a solvated environment over 10 ns. Extended FH2 FMN1 is shown in teal blue, while WW1-WW2 FNBP4 is depicted in raspberry red.
